# CDK activity provides temporal and quantitative cues for organizing genome duplication

**DOI:** 10.1371/journal.pgen.1007214

**Published:** 2018-02-21

**Authors:** Anthony Perrot, Christopher Lee Millington, Blanca Gómez-Escoda, Diane Schausi-Tiffoche, Pei-Yun Jenny Wu

**Affiliations:** Genome Duplication and Maintenance Team, Institute of Genetics and Development, CNRS UMR, Rennes, France; MRC Laboratory of Molecular Biology, UNITED KINGDOM

## Abstract

In eukaryotes, the spatial and temporal organization of genome duplication gives rise to distinctive profiles of replication origin usage along the chromosomes. While it has become increasingly clear that these programs are important for cellular physiology, the mechanisms by which they are determined and modulated remain elusive. Replication initiation requires the function of cyclin-dependent kinases (CDKs), which associate with various cyclin partners to drive cell proliferation. Surprisingly, although we possess detailed knowledge of the CDK regulators and targets that are crucial for origin activation, little is known about whether CDKs play a critical role in establishing the genome-wide pattern of origin selection. We have addressed this question in the fission yeast, taking advantage of a simplified cell cycle network in which cell proliferation is driven by a single cyclin-CDK module. This system allows us to precisely control CDK activity *in vivo* using chemical genetics. First, in contrast to previous reports, our results clearly show that distinct cyclin-CDK pairs are not essential for regulating specific subsets of origins and for establishing a normal replication program. Importantly, we then demonstrate that the timing at which CDK activity reaches the S phase threshold is critical for the organization of replication in distinct efficiency domains, while the level of CDK activity at the onset of S phase is a dose-dependent modulator of overall origin efficiencies. Our study therefore implicates these different aspects of CDK regulation as versatile mechanisms for shaping the architecture of DNA replication across the genome.

## Author summary

The duplication of the genetic material is a highly conserved and tightly regulated process that is essential for cell proliferation. DNA synthesis initiates at sites called origins that are distributed throughout the genome. Replication origins are not all used equivalently, and their patterns of activation along the chromosomes give rise to specific profiles, or programs, of DNA replication. These programs change during development and in response to external stimuli, and we have previously shown that they have important consequences for cellular function. However, we still do not understand the mechanisms by which cells establish different replication patterns. Here we investigate the role of the cyclin-dependent kinase (CDK) family of proteins, whose activities are critical for cell cycle progression, in regulating the organization of genome duplication. Taking advantage of a system that allows us to precisely modulate CDK activity levels in living cells, we demonstrate that both the temporal and quantitative controls of CDK function are crucial for determining distinct programs of DNA replication. Our work therefore uncovers a fundamental link between CDK activity, a central input in a variety of cellular and developmental processes, and the architecture of genome duplication.

## Introduction

The accurate duplication of the genetic material relies on a multilayered control of the initiation of DNA synthesis at replication origins. Origins fire at characteristic times during S phase and are activated with particular frequencies, or efficiencies, in a population of cells. Together with the distribution of origins along the chromosomes, these parameters define the genome-wide program of DNA replication. This organization of genome duplication in replication timing and efficiency domains is a conserved feature among eukaryotes, and these programs are remarkably sensitive to developmental states as well as to external stimuli [1–4]. Interestingly, although there is accumulating evidence that the spatiotemporal pattern of DNA synthesis has consequences for cellular function beyond simply duplicating the genome [4,5], we still do not understand the mechanisms by which cells establish and modulate specific replication programs.

Central to the regulation of replication initiation are members of the cyclin-dependent kinase (CDK) family, which phosphorylate key components of the pre-replicative (pre-RC) and pre-initiation (pre-IC) complexes [6–10]. CDK functions require interactions with various cyclin partners, and oscillations in CDK activity drive cell cycle progression [6,11–13]. In particular, S phase onset occurs when a low threshold of CDK activity is attained during G1 [13]. Furthermore, proper regulation of CDK is critical for origin firing and genome maintenance [14–16]. However, despite the essential functions of CDK as well as our detailed knowledge of its regulators and targets, we know surprisingly little about how it may contribute to determining the genome-wide program of DNA replication. For instance, there is conflicting evidence from a variety of systems regarding the roles of multiple cyclin-CDK pairs in regulating replication initiation. On one hand, distinct cyclin-CDK complexes appear to have non-overlapping functions for genome duplication and to modulate particular subsets of origins in organisms ranging from budding yeast to mouse [17–22]. In contrast, a number of studies have questioned the absolute requirement for specific CDKs or cyclins in sustaining genome duplication [12,13,21,23,24]. Indeed, Cdk1 is sufficient to support cell proliferation in the early mouse embryo in the absence of all interphase CDKs [24], and in budding yeast cells lacking the S phase cyclins, both early and late replication origins are fired during a delayed S phase [17]. Previous reports have also presented paradoxical outcomes of altering CDK function: both lowering CDK activity through chemical inhibition [21,25] and increasing activity via elevated cyclin levels or loss of a CDK inhibitor [14–16] result in reductions in replication initiation. Collectively, these findings highlight the complex and unresolved question of how the regulation of CDK activity, from the time it takes to reach the S phase threshold to its level at S phase entry, may shape the organization of genome duplication along the chromosomes.

In the present study, we aimed to investigate the functions of these critical parameters of CDK activity in establishing the program of DNA replication. One impediment to addressing this question *in vivo* is the presence of multiple cyclin-CDK complexes, which makes it difficult to dissociate potential qualitative differences in substrate phosphorylation provided by different cyclin-CDK pairs from quantitative changes in the dynamics and levels of overall CDK activity. To circumvent this issue, we have applied a synthetic biology approach in the fission yeast, taking advantage of a system that replaces the endogenous cell cycle circuit with a simplified CDK module [13,26]. In wild-type *Schizosaccharomyces pombe*, cell cycle progression is controlled by oscillations in CDK activity that rely on the association of CDK (Cdc2) with distinct cyclins (Cig1, Cig2, and Puc1 for G1 and S; Cdc13 for mitosis). In contrast, the synthetic CDK module consists of a fusion between Cdc13 and Cdc2 that can autonomously drive cell proliferation in the absence of all other cell cycle cyclins [13]. These “minimal” cells display no detectable phenotypes, presenting the same length of S phase and cell cycle distribution as wild-type cells [13]. Critical to our investigations, the Cdc13-Cdc2 fusion protein harbors a mutation in its Cdc2 moiety that allows for reversible and dose-dependent modulation of its kinase activity by non-hydrolyzable ATP analogs [13,27]. Importantly, previous studies using this system have demonstrated a tight quantitative relationship between CDK activity and the level of inhibitor to which cells are exposed. These notably reported differences in substrate phosphorylation [28], periodic gene expression [29], cell cycle progression [13], and cell size at division [30] that were dependent on the concentration of inhibitor applied. This powerful approach thus allows us to impose precise changes in a single CDK activity *in vivo* in order to explore the roles of the dynamics and levels of CDK activity in origin selection.

Using this unique model, we began by evaluating whether qualitative inputs from multiple cyclin-CDK complexes are required to generate a normal profile of replication initiation. Our results revealed that the regional domains of origin timing and efficiency that are established by a single cyclin-CDK activity are virtually identical to those in wild-type cells. Next, we specifically manipulated two key parameters of CDK activity: 1) the timing at which sufficient activity is available for triggering S phase entry and 2) the level of CDK activity at the G1/S transition. First, we showed that the timing when CDK activity crosses the S phase threshold is critical for regulating the organization of origin firing in distinct domains along the chromosomes. Indeed, we demonstrated that prolonging G1 phase through transient CDK inhibition leads to a genome-wide redistribution of the Cdc45 replication factor and a reprogramming of replication initiation. Second, we found that CDK function has a clear quantitative impact on origin firing throughout the genome. Cells are exquisitely sensitive to changes in CDK activity at the G1/S transition, which result in dose-dependent alterations in replication initiation that affect the entire spectrum of origin efficiencies. Collectively, our results therefore demonstrate that the temporal and quantitative controls of CDK activity provide separate inputs whose integration is crucial for determining specific genome-wide programs of DNA replication.

## Results

### Distinct cyclin-CDK complexes are not required for the establishment of a normal replication program

While there is clear redundancy between distinct cyclin-CDK combinations in providing sufficient activity to trigger S phase [13,17,23,24], evidence suggests that cyclin-CDK diversity may in fact be critical for origin selection [19,21,22]. Given these contrasting observations, we investigated the importance of operating with multiple cyclin-CDK pairs for the establishment of a wild-type profile of origin usage along the chromosomes. To this end, we used a simplified cell cycle control network in the fission yeast that consists of a fusion between the mitotic cyclin Cdc13 and the CDK Cdc2 (referred to as Cdc13-Cdc2). Oscillations in the activity of this Cdc13-Cdc2 module are sufficient to drive cell proliferation in the absence of other cell cycle cyclins, with no apparent changes in S phase onset or duration [13].

**Fig 1:**
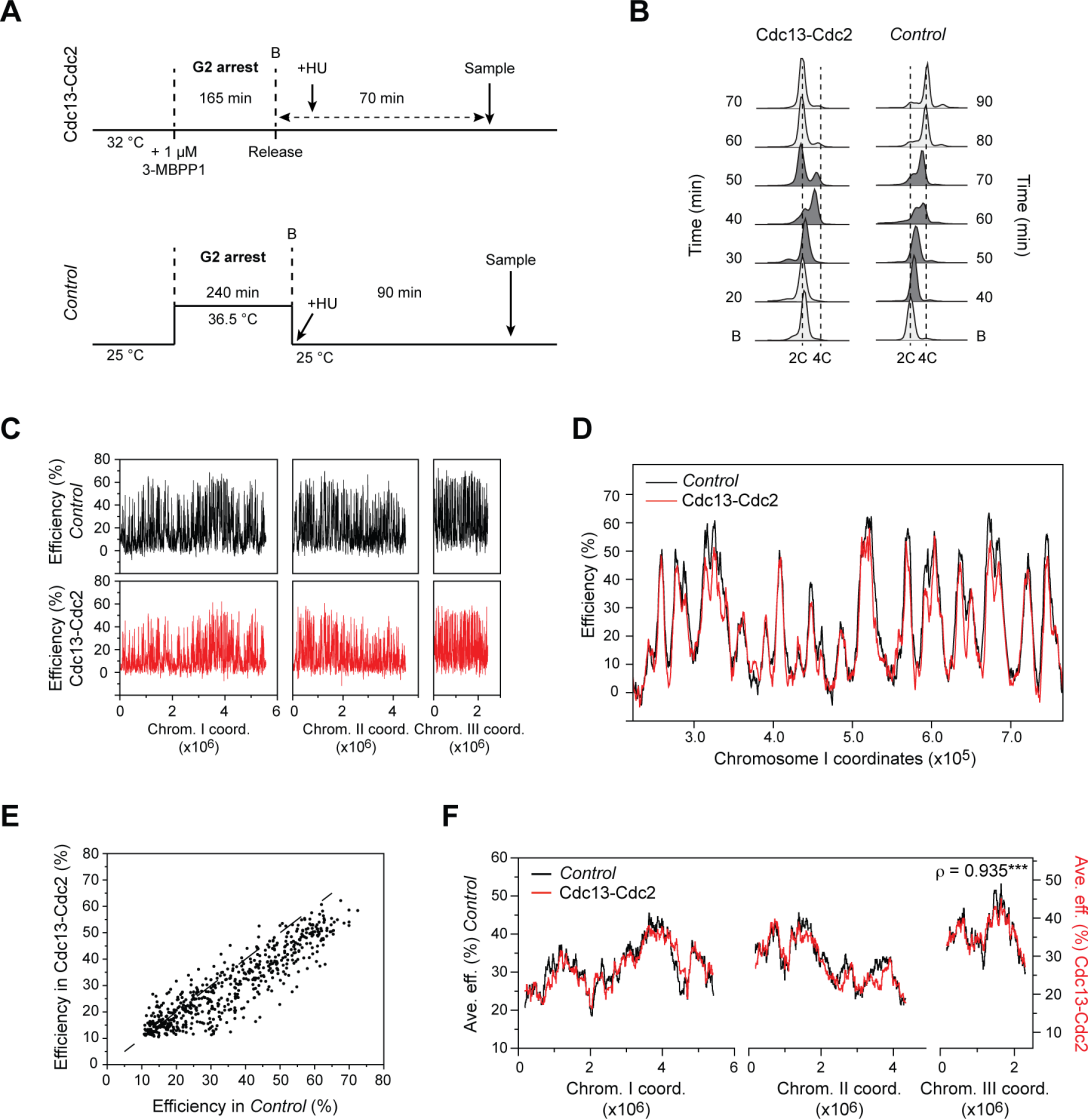
Program of DNA replication in cells operating with a single cyclin B-CDK fusion protein. **A)** Experimental design for determining the replication profiles in Cdc13-Cdc2 and *Control* cells. Top: Schematic for Cdc13-Cdc2 cells. For array experiments, 12 mM HU was added 10 min after inhibitor removal (Release). Cells were then harvested 60 min following the addition of HU (70 min after release from G2), after S phase is completed in a sample without HU (see *B*). Bottom: Schematic for *cdc25-22* cells. For array experiments, HU was added at the time of the return to permissive temperature. Cells were then harvested 90 min following the shift to 25°C, when S phase is completed in a sample without HU (see *B*). B: Block. **B)** DNA content analysis of Cdc13-Cdc2 and *Control* cells undergoing a synchronous S phase. Experimental procedures were as in *A*, but without HU treatment. These assays allowed the determination of the timing of sampling when using HU. Samples in which cells are undergoing DNA replication are shown in dark gray. Cdc13-Cdc2 cells arrested in G2 with a 2C DNA content (B: Block as in *A*) enter S phase ~30 min after release from inhibitor treatment. A 4C peak then appears, and cell division occurs while S phase is finishing, resulting in a 2C peak. *Control* cells initially arrested in G2 with a 2C DNA content (B: Block as in *A*) enter S phase at ~40 min following the release from G2. This leads to the appearance of a 4C peak, which is resolved upon cytokinesis shortly after the genome is duplicated (profiles prior to S phase and after cell division, which occurs ~100 min after release, are not shown). See Materials and Methods for additional details for the interpretation of flow cytometry profiles. **C**) Origin usage profiles of *Control* (black) and Cdc13-Cdc2 (red) cells. x-axis: chromosome coordinates, y-axis: origin efficiencies. For full detailed profiles, see S1A Fig. **D)** Detailed view of origin efficiencies in a representative region of the genome for *Control* (black) and Cdc13-Cdc2 (red) cells. Data are as in *C*. x-axis: chromosome coordinates, y-axis: origin efficiencies. **E)** Comparison of origin efficiencies in Cdc13-Cdc2 vs. *Control* cells. The efficiencies of the 598 origins common to both replication programs are shown. x-axis: efficiency in the *Control* program, y-axis: efficiency in the Cdc13-Cdc2 program. The dashed line represents efficiencies if they were identical in the two backgrounds. **F)** Regional profiles of replication domains in *Control* (black, scale on left y-axis) and Cdc13-Cdc2 (red, scale on right y-axis) cells. The averages of origin efficiencies were determined for continuous windows of ~250 kb (1000 probes) across the genome. x-axis: chromosome coordinates, y-axis: average origin efficiencies. Note that for comparison, the Cdc13-Cdc2 profile is shown on a scale that is shifted to adjust for the 4.2% difference in overall average origin efficiency between the two backgrounds (see S1B Fig). This does not alter the absolute efficiency scales on the y-axes, which each span 50% efficiency. Replication efficiency domains are clearly apparent: for instance, the right arm of chromosome II (2.5–3.5 Mb) comprises a low efficiency region, whereas the right arm of chromosome I (3–4 Mb) represents a high efficiency region. The Spearman’s rank correlation coefficient (ρ) comparing the replication programs in the two backgrounds is shown and demonstrates a very strong correlation. ***: p-value < 0.001.

We first assessed the consequences of undergoing S phase in the absence of the G1/S cyclins Cig1, Cig2, and Puc1, comparing the genome-wide pattern of origin usage in Cdc13-Cdc2 cells with that of cells containing the full complement of cyclins (referred to as *Control*). For these experiments, we determined the program of origin selection in each background by synchronizing cells in G2 and allowing them to progress through mitosis and enter S phase in the presence of 12 mM hydroxyurea (HU). HU limits the extent of replication around the sites of initiation, permitting the identification of origins and an assessment of their efficiencies [4,31]. For Cdc13-Cdc2 cells, G2 arrest was achieved by addition of 1 μM of the ATP analog 3-MBPP1 for 2 h 45 min at 32°C [13]. The inhibitor was then washed off to induce cells to synchronously re-enter the cycle (Fig 1A, top panel and Fig 1B, left panel; see also [13]). *Control* cells were synchronized using the *cdc25-22* temperature sensitive mutation [32]: cells were shifted to the non-permissive temperature of 36.5°C for 4 h, followed by a return to permissive temperature (25°C), resulting in a resumption of the cell cycle (Fig 1A, bottom panel and Fig 1B, right panel). Genomic DNA was isolated prior to the release from G2 arrest (unreplicated DNA) and during S phase in HU-treated cells. For the latter, samples were collected at a time when bulk S phase is complete in the absence of HU (Fig 1B). Replication origins were then mapped by competitive hybridization of differentially-labelled G2 and S phase samples to microarrays containing probes that cover the fission yeast genome [4]. Initiation sites were identified as peaks of increased copy number in the S phase samples, with the amplitude of the signal reflecting origin efficiency (for instance, a copy number of 1.1 represents 10% efficiency) [4,31]. This method has been previously validated [4,31] and generates profiles that are similar to those obtained with other approaches [33,34].

Remarkably, our results revealed highly comparable genome-wide profiles of origin usage in *Control* and Cdc13-Cdc2 cells (Fig 1C and 1D, S1A Fig). Using a 10% efficiency cutoff, we identified similar numbers of origins, with over 91% of these sites being shared between the two programs (S1B Fig, S1 Table). We then asked whether the absence of G1 and S cyclins specifically alters the efficiencies of particular origin subsets, as suggested by previous studies [17,21,22]. While we found a modest average difference of 4.2% in origin usage, this effect was observed across the entire range of origin activities (Fig 1E). This pointed to a slight overall reduction in efficiencies in Cdc13-Cdc2 cells, rather than a targeted alteration of specific sites. Strikingly, regional analyses of origin usage assessing average efficiencies in continuous ~250 kb windows showed that the domains of low and high origin activity in Cdc13-Cdc2 are virtually identical to those in the *Control* (Fig 1F). Our results therefore indicate that cyclin diversity is not required for the activation of specific groups of origins. Moreover, we provide the first demonstration, to our knowledge, that a single cyclin-CDK pair is sufficient for establishing an organization of replication initiation that is almost indistinguishable from that produced by the full complement of cyclins. Importantly, these data show that the Cdc13-Cdc2 background represents an ideal system for investigating the consequences of modulating CDK activity on the program of genome duplication.

### Temporal regulation of CDK activity is a critical determinant of the regional organization of DNA replication

**Fig 2:**
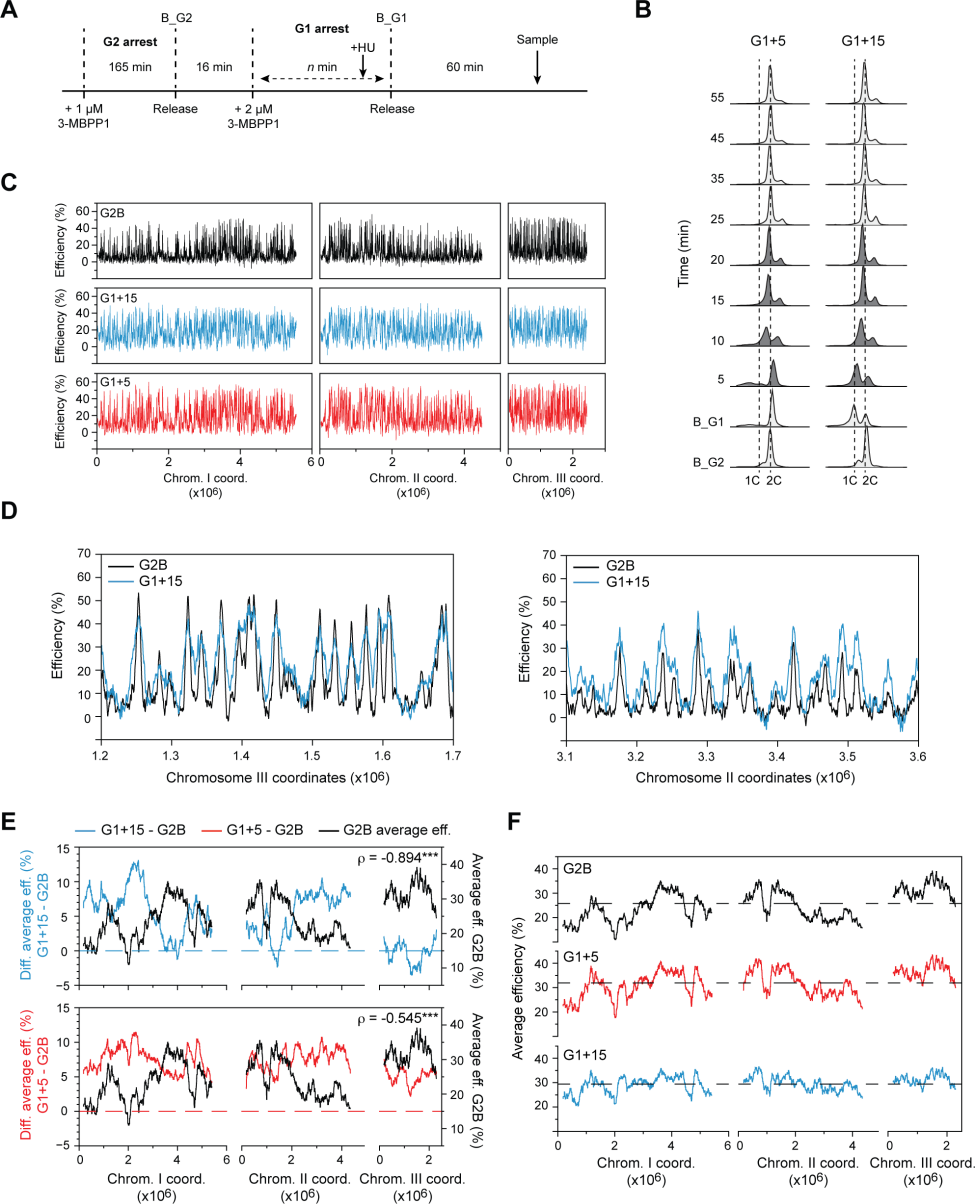
The timing of CDK availability for the G1/S transition modulates the regional organization of genome duplication. **A)** Experimental design for determining the replication profiles in Cdc13-Cdc2 cells with a delay in CDK availability. *n* represents 20 min (G1+5) or 30 min (G1+15). For array experiments, HU was added to the cells 10 min before the release from the 2 μM 3-MBPP1 inhibitor treatment (B_G1). Cells were harvested 5 min before (unreplicated DNA) and 60 min after this release, after S phase is completed in a sample without HU (see *B*). B_G2: G2 block. B_G1: G1 block. **B)** DNA content analysis of G1+5 and G1+15 cells undergoing a synchronous S phase. Experimental procedures were as in *A*, but without HU treatment. Samples in which cells are undergoing DNA replication are shown in dark gray. B_G2 and B_G1 are as in *A*. G1+5 cells are initially arrested in G2 with a 2C DNA content (B_G2), and they maintain this DNA content during the 2 μM inhibitor treatment that extends G1 (B_G1). Cells begin replicating by 5 min after the release from G1, leading to a rightward shift in the peak. By 10 min, cytokinesis has started to occur, concomitant with replication, resulting in the presence of both 2C and 4C peaks. G1+15 cells are initially arrested in G2 with a 2C DNA content (B_G2). In contrast to the G1+5 condition, they begin to divide during the additional 10 min of the G1 arrest (B_G1), giving rise to a 1C peak. Cells enter S phase by 5 min after the release from G1 arrest, resulting in a rightward shift of the peaks. See Materials and Methods for additional details for the interpretation of flow cytometry profiles. **C)** Origin usage profiles of Cdc13-Cdc2 cells released from G2 (G2B) or undergoing short G1 extensions (G1+15 and G1+5). Note that all origin efficiency profiles were assessed in 24 mM HU (see Materials and Methods for more details). x-axis: chromosome coordinates, y-axis: origin efficiencies. For full detailed profiles and one-to-one comparisons of origin efficiencies, see S2A and S2B Figs. **D)** Detailed views of origin efficiencies in representative regions of the genome for G2B (black) and G1+15 (blue). x-axis: chromosome coordinates, y-axis: origin efficiencies. **E)** Regional analyses of the changes in origin efficiencies between the G2B, G1+15, and G1+5 replication programs. Top panel: Differences of the average origin efficiencies (G1+15 - G2B) determined in continuous windows of 1000 probes (~250 kb) (blue, scale on left y-axis). The profile of average origin efficiencies in G2B is indicated in black (scale on right y-axis). Bottom panel: Differences of the average origin efficiencies (G1+5 - G2B) determined in continuous windows of 1000 probes (~250 kb) (red, scale on left y-axis). The profile of average origin efficiencies in G2B is indicated in black (scale on right y-axis). Dashed lines represent 0% efficiency changes. x-axis: chromosome coordinates. The Spearman’s rank correlation coefficients (ρ) comparing the differences in origin usage vs. average origin efficiencies in G2B are indicated. This shows very strong and moderate negative correlations for G1+15 - G2B vs. G2B and G1+5 - G2B vs. G2B, respectively. ***: p-value < 0.001. **F)** Regional profiles of replication domains in G2B (black), G1+5 (red), and G1+15 (blue). The averages of origin efficiencies were determined for continuous windows of ~250 kb (1000 probes). Dashed lines indicate the average efficiency of all of the origins in each program (G2B: 26.4%; G1+5: 31.9%; G1+15: 29.4%). Data for the G2B conditions are as in *E*. x-axis: chromosome coordinates, y-axis: average origin efficiencies.

We next determined the impact of the temporal regulation of CDK on the replication program, ascertaining if the timing at which CDK activity reaches the S phase threshold plays a role in origin selection. To this end, Cdc13-Cdc2 cells initially arrested in G2 using 1 μM 3-MBPP1 were released to synchronously re-enter the cell cycle. After mitotic exit, these cells were exposed to 2 μM 3-MBPP1 for increasing periods of time (Fig 2A): this concentration of inhibitor reduces CDK activity to a sufficiently low level to extend G1 and delay the onset of DNA replication (Fig 2B). Cells were then released from this transient G1 arrest upon removal of the inhibitor and allowed entered S phase in the presence of HU. To assess the programs of origin efficiency associated with prolonged G1 phases, genomic DNA from the HU-treated cells in S phase was then competitively hybridized against unreplicated DNA samples collected just before the release from G1 (see Fig 2B and the Materials and Methods section for details on the timings). Note that for these and all subsequent origin mapping experiments, we used 24 mM HU to prevent further extension of DNA synthesis around initiation sites. As a control for these conditions, we determined origin usage in Cdc13-Cdc2 cells synchronized in G2 as in Fig 1A but treated with 24 mM HU upon release (referred to as G2B); these data served as the reference for all analyses presented below.

Our results uncovered striking changes in the profile of replication initiation upon alteration of the timing at which CDK activity crosses the S phase threshold (Fig 2C and S2A Fig). First, we observed that increasing the length of G1 by 15 min led to both increases and decreases in origin usage (S2B Fig, left panel) in different regions of the genome (Fig 2D; note that 15 min corresponds to a doubling of the duration of G1, see Materials and Methods). To determine whether these changes in origin selection are specific to particular chromosomal domains, we compared the regional efficiency profile of G1+15 with that of cells released from G2 and progressing through a normal G1 (G2B), assessing the differences in origin efficiencies in continuous ~250 kb windows across the genome. This identified a signature alteration of the replication program: origin activity was increased in regions of low efficiency, while origin usage in efficient domains was unchanged or modestly reduced (Fig 2E, top panel). Even a 5 min extension of G1 displayed a reduction in the differences between high and low efficiency domains, although to a lesser extent (G1+5; Fig 2E, bottom panel and S2B Fig, right panel). Consistent with these observations, we found strong negative correlations between the replication program in G2B and the changes in origin efficiencies that result from an extended G1 (Fig 2E). Finally, to confirm our findings and rule out the possibility that these alterations in origin selection were due to differences in the duration of HU exposure between G2B and the G1 extensions, we ascertained origin usage in cells as for the G2B condition but maintained for an additional 30 min in HU (see Materials and Methods for details). Our data established that origin efficiencies are not affected by the length of the HU treatment (S2C Fig; see also S2 Table), confirming that a prolonged G1 phase leads to distinct changes in origin usage.

All together, our results demonstrate a gradual equalization of origin activities between genomic regions when G1 is extended (Fig 2F). Our findings thus provide evidence that the replication program is extremely responsive to delays in CDK function and that even modest changes in the timing of CDK availability have significant effects on the organization of DNA replication.

### G1 extension results in a genome-wide redistribution of Cdc45 recruitment

**Fig 3:**
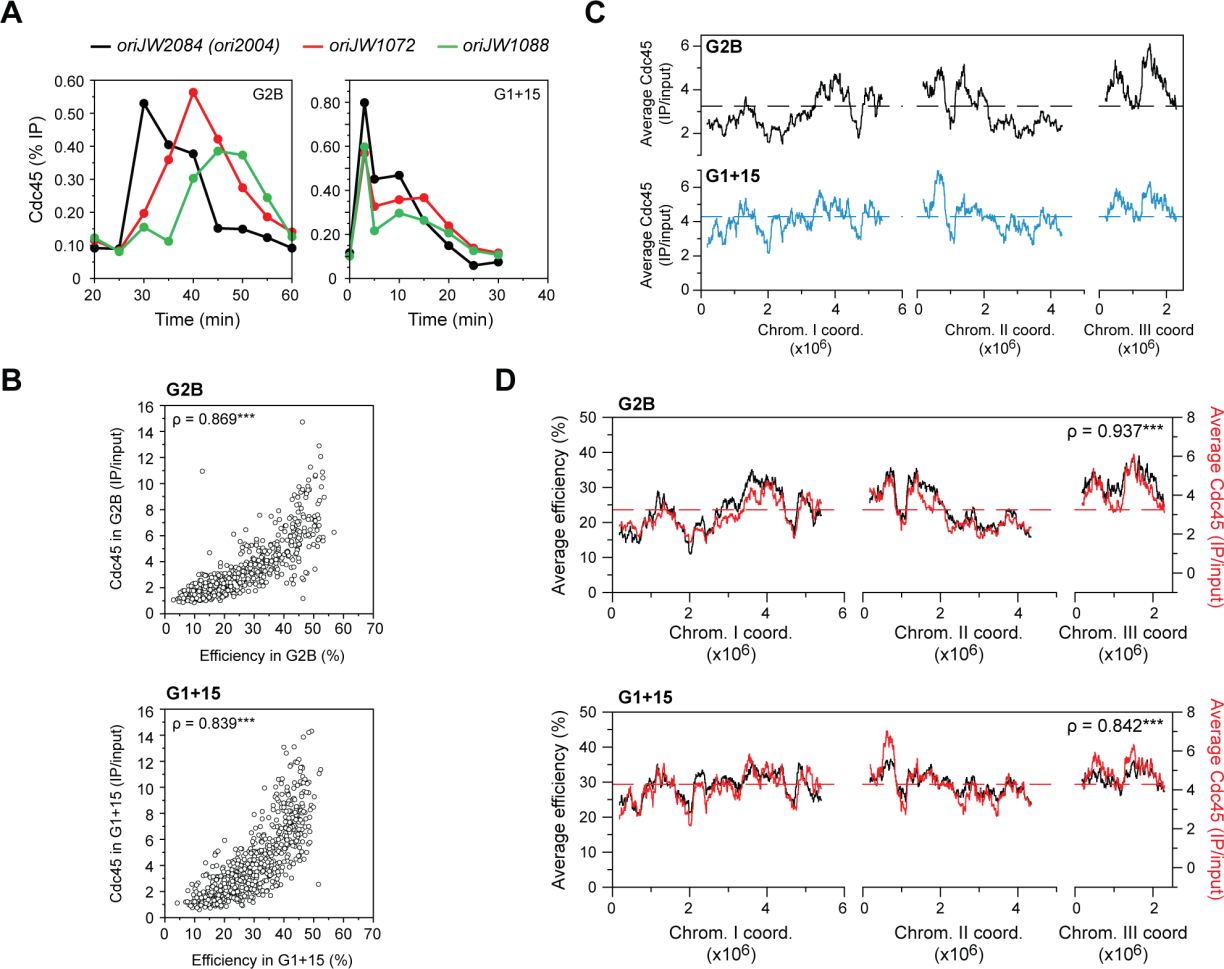
G1 extension results in a redistribution of Cdc45 binding. **A)** Time courses of chromatin immunoprecipitation of Cdc45 in the G2B (left panel) and G1+15 (right panel) conditions (see Figs 1A and 2A for the experimental procedures). Note that these experiments were performed in the absence of HU to allow progression through S phase. Efficiencies of the origins analyzed are as follows: in G2B, *oriJW2084* (*ori2004*): 45%, *oriJW1072*: 21%, *oriJW1088*: 21%; in G1+15, *oriJW2084* (*ori2004*): 38%, *oriJW1072*: 29%, *oriJW1088*: 36%. x-axis: time after release from G1 arrest; y-axis: % IP. *n* = 2, a representative experiment is displayed. **B)** Comparison of Cdc45 recruitment with origin efficiency at each origin. Top panel: G2B; bottom panel: G1+15. Detailed genome-wide profiles of Cdc45 binding are displayed in S3 Fig. We observe a strong correlation between Cdc45 occupancy levels and origin efficiencies in both conditions. x-axis: origin efficiencies; y-axis: Cdc45 binding. Each circle represents an origin. The Spearman’s rank correlation coefficients (ρ) are displayed. ***: p-value < 0.001. **C)** Regional profiles of Cdc45 recruitment in G2B (black) and G1+15 (blue). The averages of Cdc45 binding at origins were determined for continuous windows of ~250 kb (1000 probes) across the genome. x-axis: chromosome coordinates, y-axis: average Cdc45 binding (IP/input). Dashed lines indicate the average Cdc45 level at all origins in each condition. **D)** Comparisons of the regional profiles of origin efficiency (black, left y-axis; profiles as in Fig 2F) and Cdc45 binding (red, right y-axis; profiles as in *C*) in G2B (top panel) and G1+15 (bottom panel). The Spearman’s rank correlation coefficients (ρ) comparing the profiles of origin usage vs. Cdc45 binding are indicated and reveal very strong positive correlations ***: p-value < 0.001. Dashed lines for genome-wide average Cdc45 levels are as in *C*.

We and others have previously shown that the timing and efficiency of origin usage is regulated by the recruitment of limiting initiation factors [35–37]. Therefore, in light of our results above, we evaluated the impact of delaying the availability of CDK activity on the formation of the pre-initiation complex. Cdc13-Cdc2 cells undergoing a synchronous S phase in the absence of HU after G2 arrest (G2B) or upon release from a 15 min G1 extension (G1+15) were sampled from G1 through S phase, and the binding of the pre-IC component Cdc45 was assessed. For this assay, we tested three representative origins: *ori2004* (referred to as *oriJW2084* in S1 Table), an efficient origin in G2B (45%) and G1+15 (38%), as well as *oriJW1072* and *oriJW1088*, which are inefficient origins in G2B (both 21%) that become more efficient in G1+15 (29% and 36%, respectively). As expected, in cells undergoing a synchronous S phase after release from G2 arrest, we observed that Cdc45 binds to the efficient origin *ori2004* at an earlier time than to the inefficient origins *oriJW1072* and *oriJW1088* (Fig 3A, left panel). However, this was not the case in the G1+15 condition, where Cdc45 bound similarly to all three origins (Fig 3A, right panel). These data suggest that extending G1 results in a redistribution of limiting replication components.

We then asked whether these alterations in pre-IC formation occur throughout the genome and contribute to the changes in efficiencies that are observed after a G1 extension. To this end, we performed chromatin immunoprecipitation followed by microarray analysis (ChIP-chip) of Cdc45 in the G2B and G1+15 conditions. For these experiments, cells were collected at the G1/S transition, when Cdc45 recruitment reaches a maximum at early-firing origins (Fig 3A). First, we observed that Cdc45 is bound at initiation sites in both situations (S3A–S3D Fig) and that the levels of Cdc45 at origins are strongly correlated with origin efficiencies (Fig 3B). Next, we performed regional analyses of Cdc45 binding and demonstrated that there is a striking equalization of Cdc45 recruitment between distinct genomic regions (Fig 3C and S3E Fig): the differences in the levels of Cdc45 at origins between replication domains are reduced, similar to what we observed for efficiencies. This is also illustrated in S3F Fig, which shows very different distributions for the deviations of the regional profiles of origin efficiencies and Cdc45 from their corresponding means in G1+15 compared to G2B. Furthermore, we established a remarkable correspondence between the regional profiles of origin efficiencies and Cdc45 binding (Fig 3D), with Spearman’s rank correlation coefficients that showed very strong positive relationships between these parameters in both the G2B and G1+15 conditions. Our results therefore indicate that the temporal control of CDK activity alters the competition between initiation sites for limiting replication factors, thereby regulating pre-IC assembly and subsequent origin usage.

### Limits of reorganizing replication initiation through G1 extension

**Fig 4:**
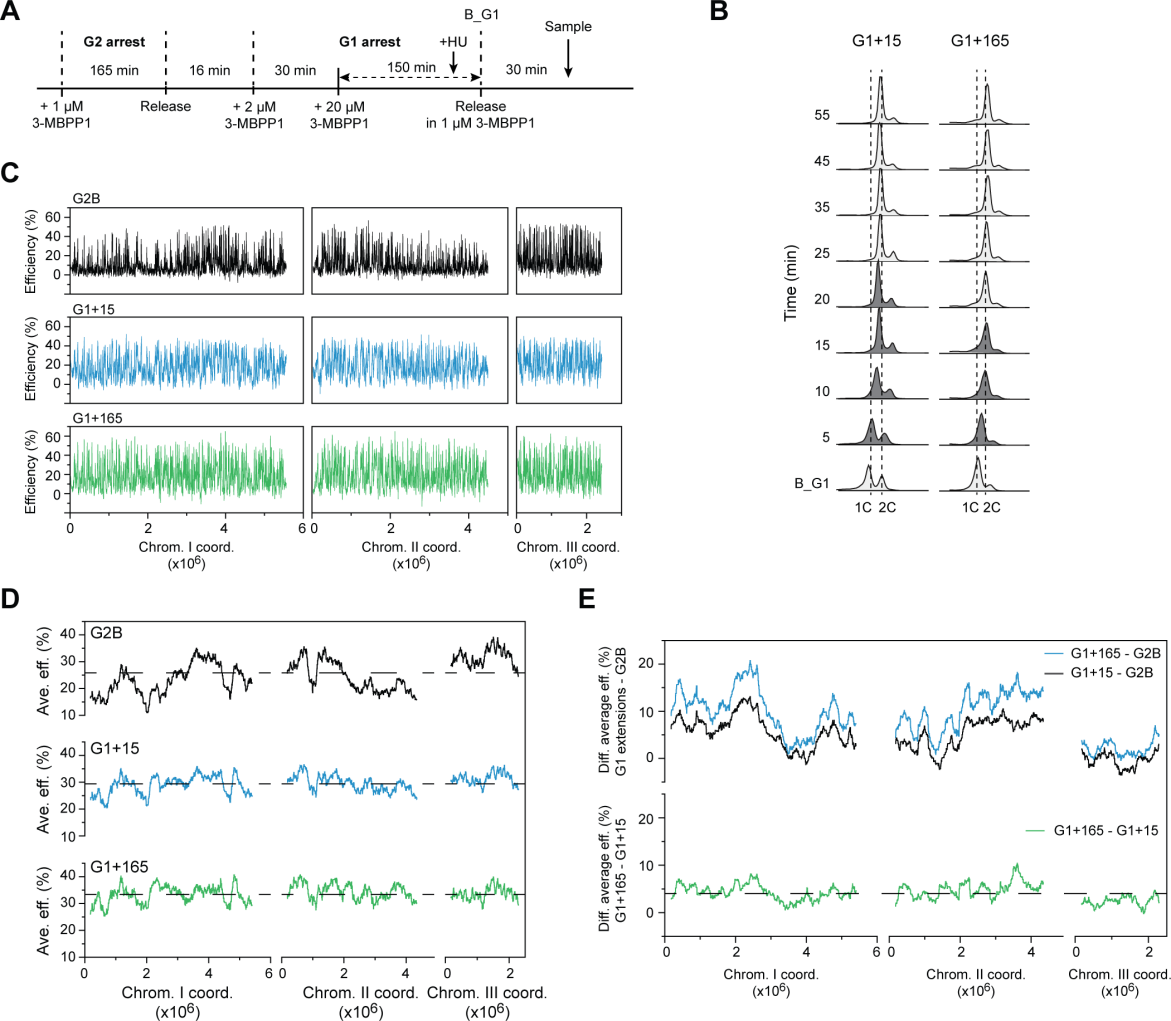
Limits of reprogramming DNA replication by temporal regulation of CDK activity. **A)** Experimental design for determining the replication profile in Cdc13-Cdc2 cells with a long delay in CDK availability (G1+165). For array experiments, HU was added to the cells 10 min before the release from the 20 μM 3-MBPP1 inhibitor treatment (B_G1). Cells were harvested 30 min following this release, after S phase is completed in a sample without HU (see *B*). B_G1: G1 block. **B)** DNA content analysis of G1+15 and G1+165 cells undergoing a synchronous S phase. The data for G1+15 are as in Fig 2B. The experimental procedure for G1+165 was as in *A*, but without HU treatment. Samples in which cells are undergoing DNA replication are shown in dark gray. B_G1 indicates the end of the prolonged G1. In the G1+165 conditions, cells have fully divided by the time of the release (B_G1), resulting in one major 1C peak. The ensuing S phase leads to a rightward shift of this peak to 2C. See Materials and Methods for additional details for the interpretation of flow cytometry profiles. **C)** Origin usage profiles of G2B, G1+15, and G1+165. Data for G2B and G1+15 are as in Fig 2C. x-axis: chromosome coordinates, y-axis: origin efficiencies. For full detailed profiles and one-to-one comparisons of origin efficiencies, see S4A and S4B Fig, respectively. **D)** Regional profiles of replication domains in G2B (black), G1+15 (blue), and G1+165 (green). The averages of origin efficiencies were determined for continuous windows of ~250 kb (1000 probes). Data for G2B and G1+15 are as in Fig 2F. Dashed lines indicate the average efficiencies of all the origins in each program (G2B: 24.9%; G1+15: 29.4%; G1+165: 33.4%). x-axis: chromosome coordinates, y-axis: average origin efficiencies. **E)** Regional analysis of the changes in origin efficiencies. Top panel: Differences of the average origin efficiencies (blue: G1+165 - G2B; black: G1+15 - G2B) determined in continuous windows of 1000 probes (~250 kb). Bottom panel: Differences of the average origin efficiencies (green: G1+165 - G1+15) determined in continuous windows of 1000 probes (~250 kb). The dashed line marks the average difference in origin efficiencies between the G1+165 and G1+15 conditions (4%). x-axis: chromosome coordinates, y-axis: difference in average origin efficiencies. The higher efficiencies in G1+165 are likely to be due to the higher CDK activity level in this condition (see Fig 5).

As short delays in the timing of CDK availability (5 and 15 min in our assays) are sufficient to induce alterations in the genome-wide profile of origin usage, we next addressed the extent to which an increase in G1 length can affect the replication program. For this experiment, we modified the procedure for the CDK delay conditions described above (Fig 2A) and treated a synchronized population of Cdc13-Cdc2 cells that have undergone mitotic exit with 2μM 3-MBPP1 for 30 min, followed by further incubation with 20 μM 3-MBPP1 (Fig 4A). This enabled us to keep CDK activity below the S phase threshold for a much longer period of time. Cells were maintained in these conditions for a total of 180 min after mitotic exit, during which replication did not occur, and then released from this high concentration of 3-MBPP1 (Fig 4B; this represents a 165 min extension of G1, referred to as G1+165). As previously characterized, the Cdc13-Cdc2 protein accumulates during a long G1 arrest, and rapid change to inhibitor-free medium leads to simultaneous entry into S and M phases due to the rapid increase of CDK activity to mitotic levels [13]. To prevent this lethal phenotype, we triggered cells cycle re-entry in our assay by switching from 20 μM to 1 μM 3-MBPP1, which permits S phase onset while inhibiting mitosis. We emphasize that in all of the following experiments, cells undergo S phase without subsequent mitotic entry. To assess origin efficiencies, the cells released from this long G1 extension were allowed to enter S phase in the presence of HU. Genomic DNA from cells collected before the release from G1 was then hybridized against that from the HU-treated cells in S phase (see Fig 4B and the Materials and Methods section for details on the timing of sampling).

Intriguingly, our data demonstrate that even with a substantially longer delay of 165 min in S phase onset, the pattern of origin efficiencies along the chromosomes was similar to that of G1+15 (Fig 4C and S4A Fig). Regional analyses of the changes in replication induced by the G1+165 vs. G1+15 conditions showed a comparable reorganization of genome duplication (Fig 4D), with only a slightly greater equalization between efficiency domains and higher overall efficiencies in G1+165 (Figs 4E and S4B and S4C). In these conditions, we also observed an alteration in Cdc45 recruitment to representative origins, similar to what we found for G1+15 (S4D Fig). These data indicate that the dramatic reprogramming of DNA replication that results from regulating the timing of CDK activity is largely completed in a short period of time, implying a high degree of versatility in generating different replication profiles upon relatively limited alterations in G1 length.

Collectively, our results demonstrate that the timing at which CDK activity reaches the threshold for S phase entry is a sensitive parameter that can be finely modulated to induce major changes in the regional organization of DNA replication.

### CDK activity is a quantitative, dose-dependent regulator of origin efficiencies

**Fig 5:**
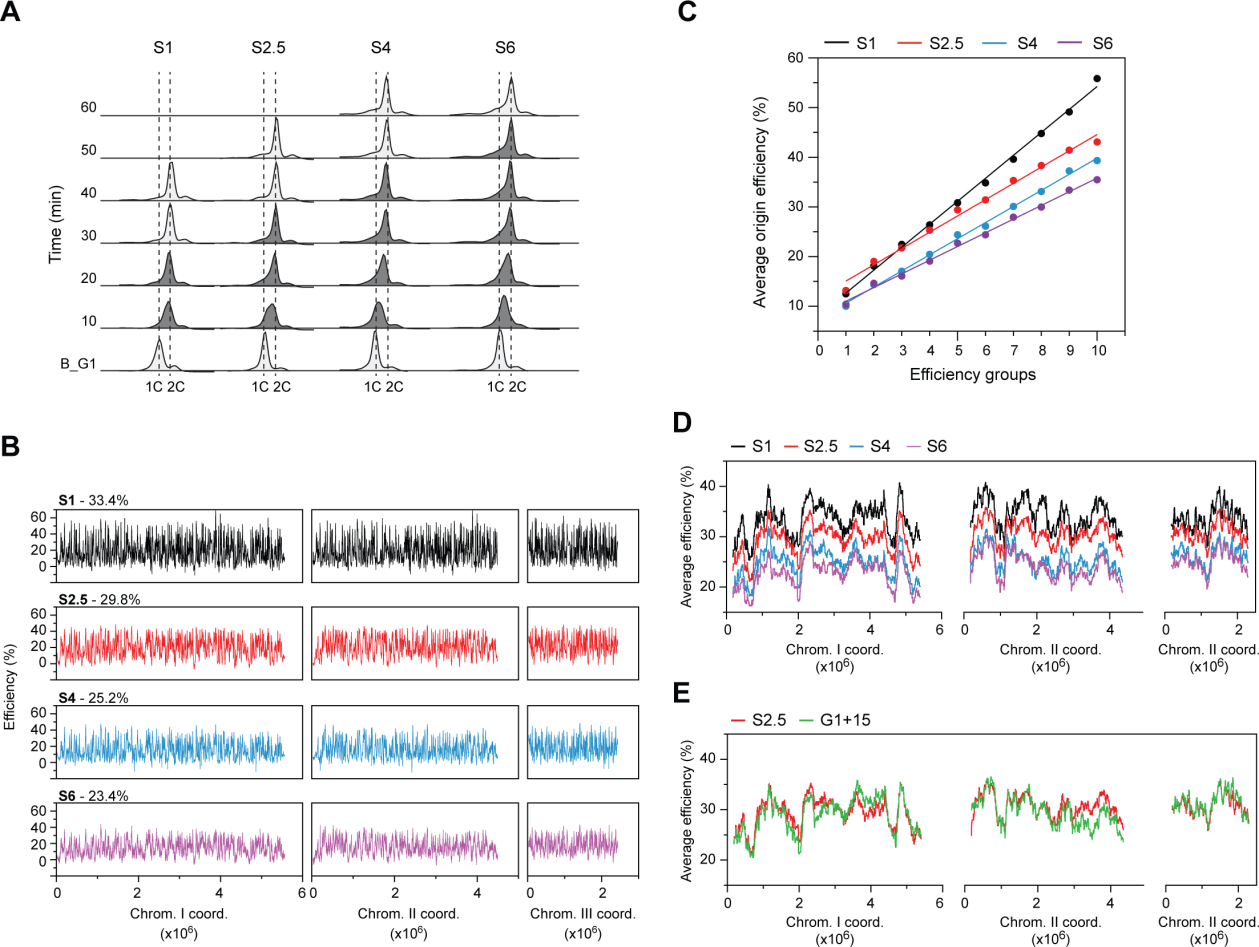
CDK activity is a dose-dependent regulator of genome-wide origin efficiencies. **A)** DNA content analysis of cells entering S phase with different concentrations of the 3-MBPP1 inhibitor. The experimental procedure was as in Fig 4A, except that cells were not treated with HU and were released into different concentrations of 3-MBPP1 after the 165 min G1 arrest (B_G1 as in Fig 4A). The 3-MBPP1 concentrations used were: S1–1 μM (data as for G1+165 in Fig 4); S2.5–2.5 μM; S4–4 μM; S6–6 μM. Samples in which cells are undergoing DNA replication are shown in dark gray. As mentioned for Fig 4A, the majority of the cells at the end of the 165 min G1 arrest have a 1C DNA content. Increasing concentrations of the inhibitor lead to prolongations of bulk S phase duration. See Materials and Methods for additional details for the interpretation of flow cytometry profiles. **B)** Origin usage profiles of S1 (black, data as in Fig 4C), S2.5 (red), S4 (blue), and S6 (purple). Experiments were performed as in Fig 4A (in the presence of HU) with release into the different inhibitor concentrations. Cells were harvested after S phase is completed in a sample without HU; this timing differs for each inhibitor treatment (see *A* and Materials and Methods). The overall average origin efficiencies for each condition are shown. x-axis: chromosome coordinates, y-axis: origin efficiencies. For full detailed profiles and one-to-one comparisons of origin efficiencies, see S5A and S5B Fig, respectively. **C)** Analysis of efficiencies in the different replication programs across the spectrum of origin activities. Origins were divided into 10 subsets containing similar numbers of origins based on their efficiencies in the S1 condition. Each point represents the average efficiency for each group. The line of best fit is shown for each CDK activity level. **D)** Regional profiles of replication domains in S1, S2.5, S4, and S6. The averages of origin efficiencies were determined in each condition for continuous windows of ~250 kb (1000 probes). Data for S1 are as in Fig 4D. x-axis: chromosome coordinates, y-axis: average origin efficiencies. **E)** Comparison of the replication domain profiles of G1+15 (data as in Fig 4D) and S2.5 (data as in *D)*. The averages of origin efficiencies were determined for continuous windows of ~250 kb (1000 probes). x-axis: chromosome coordinates, y-axis: average origin efficiencies. Overall average efficiency of origins in G1+15: 29.4%, S2.5: 29.8%. For one-to-one comparisons of origin efficiencies, see S5E Fig.

Finally, we ascertained the quantitative effect of CDK activity on the replication program. Our unique system enables us to uncouple the timing of CDK function from its activity level, inducing cells to initiate DNA synthesis with a range of CDK activities while maintaining the same G1 length. This contrasts with previous studies in which increasing CDK activity during G1 did not necessarily result in greater activity at the start of S phase but rather in advanced S phase entry [14,16]. For our experiments, origin usage was assessed in cells released from a 165 min G1 arrest (as in Fig 4A) into different concentrations of the 3-MBPP1 inhibitor (1, 2.5, 4, and 6 μM; these will be referred to as S1—the same as G1+165, S2.5, S4, and S6). Note that the higher the inhibitor concentration, the lower the CDK activity. As mentioned earlier, these conditions result in S phase entry but do not permit mitosis, allowing us to evaluate origin efficiencies in cells that undergo DNA replication with different levels of CDK activity. First, we observed that while S phase onset occurred at similar times in all conditions, decreasing CDK activity was associated with a progressive extension of S phase duration (Fig 5A). For origin efficiency assessments, cells were then released from G1 arrest as above but in the presence of HU. Unreplicated DNA from samples collected before G1 release were then hybridized against those from the HU-treated cells in S phase (see Fig 5A and the Materials and Methods section for details on the timing of sampling in the different conditions).

Our genome-wide studies of replication initiation in these assays showed that origin efficiencies are highly responsive to the levels of CDK activity: lowering this activity at the G1/S transition led to a dose-dependent reduction in overall origin usage (Figs 5B and S5A). Importantly, we demonstrated that replication initiation across the entire range of efficiencies was affected (Figs 5C and S5B), with progressively greater reductions as CDK activity is further inhibited (S5C Fig). These findings are supported by DNA combing experiments that showed an increase in interorigin distances in S6 compared to S1, consistent with the decrease in origin efficiencies (S5D Fig). Finally, in contrast to the effects of altering the timing of CDK function, all chromosomal regions responded similarly to the changes in CDK activity levels, and the replication pattern along the chromosomes was maintained (Fig 5D). We thus conclude that the level of CDK activity at S phase onset is a direct and quantitative regulator of absolute origin efficiencies throughout the genome.

All together, our results imply that specific replication programs are brought about by a combination of 1) the timing at which CDK activity triggers S phase onset and 2) the level of CDK activity at this critical transition. Independent modulation of these two parameters could therefore allow for the generation of a spectrum of replication patterns. Conversely, the same profile may be produced by different combinations of CDK timing and activity. To test this possibility, we compared the replication program after a short G1 prolongation (G1+15) with that obtained by modulating the level of CDK activity at G1/S after an extended G1 delay (in particular S2.5). Our analysis showed that origin efficiencies in these two conditions are highly comparable (S5E Fig) and that the regional replication profiles are virtually identical (Fig 5E), despite the vastly different experimental setup. This observation raises the intriguing possibility that depending on the environmental and physiological conditions, similar programs may be achieved by integrating different quantitative and temporal settings for CDK activity.

## Discussion

**Fig 6:**
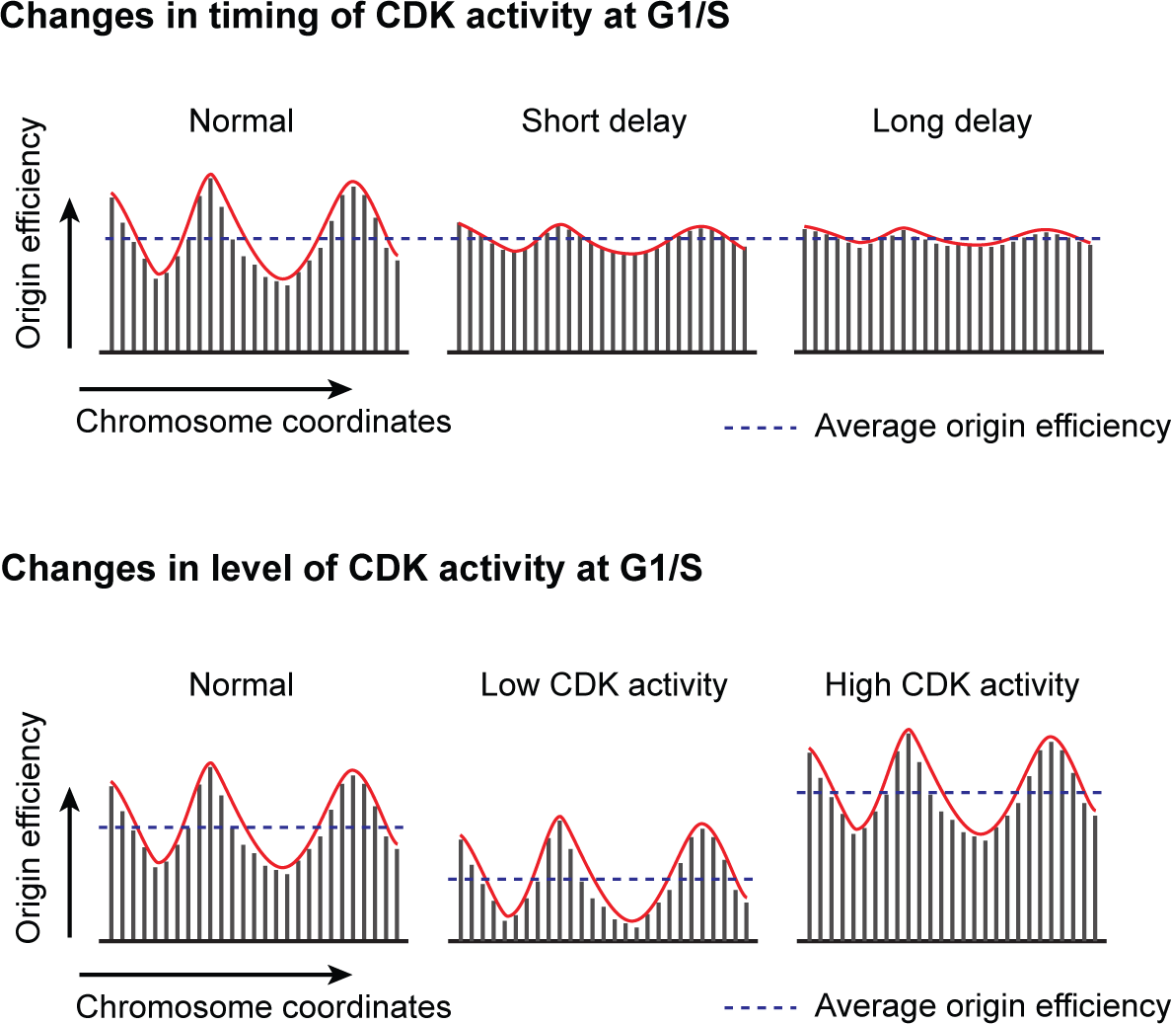
Diagram illustrating the regulation of the replication program through modulation of CDK activity. Dashed lines: average efficiencies of all of the origins in each program; red lines: regional efficiency profiles of origin usage. Top panel: Changes in the timing at which CDK activity reaches the S phase threshold result in reprogramming of replication efficiency domains. Our results show that a major equalization of origin efficiencies occurs as a consequence of short G1 extensions, while a prolonged delay leads to a minor further alteration of the replication pattern. Bottom panel: Alterations in the level of CDK activity at the G1/S transition generate quantitative changes in origin efficiencies across the entire genome without affecting the domains of efficiencies. In this case, average origin efficiencies vary depending on activity levels.

While CDK activity is undoubtedly the essential driver of cell cycle progression, remarkably little is known about how the regulation of this activity may affect the organization of genome duplication along the chromosomes. In this study, we have investigated the impact of two key features of CDK function—when it triggers S phase entry and how much activity is present at the onset of S phase—on the genome-wide program of DNA replication. Using a unique system in the fission yeast, we first show that different combinations of cyclin-CDK pairs are not required to regulate particular groups of origins and that a single qualitative CDK activity is sufficient to produce a normal profile of origin usage. Importantly, we then take advantage of this model to demonstrate that the timing at which CDK activity crosses the S phase threshold is a critical input for the organization of replication in distinct efficiency domains along the chromosomes (Fig 6, top). Notably, we find that prolonging the length of G1 induces alterations in pre-IC formation that are linked to an equalization in origin usage between genomic regions. Next, our results establish that the level of CDK activity as cells enter S phase is an extremely sensitive dose-dependent modulator of genome-wide origin efficiencies (Fig 6, bottom). Our work therefore provides evidence for CDK regulation as a versatile mechanism by which cells program DNA replication, uncovering a fundamental link between the central player in cell cycle progression and the architecture of genome duplication.

Our findings imply that the timing and level of CDK activity can be independently controlled to achieve a broad spectrum of origin usage profiles. Although we have modulated these parameters through the use of a targeted CDK inhibitor in our experiments, such regulation in a natural context may be conferred in part by cyclin and CDK diversity. Thus, the presence of multiple cyclin-CDK pairs, which have different expression, degradation, and activation programs [6], may in fact provide the precise timing and overall CDK activity levels that shape DNA replication. Our model would therefore reconcile the contrasting observations from previous reports regarding the requirement for multiple cyclin-CDK pairs in origin selection [17–22]: the specific effects of distinct cyclin-CDK combinations described in these studies may result from changes in the overall timing and level of CDK function rather than from qualitative differences in the phosphorylation of particular substrates by non-redundant complexes. Interestingly, the natural diversity in cyclin-CDKs may provide flexibility to produce complex activity profiles, leading to context-specific modulations of DNA replication. This may for instance enable cells to rapidly adapt to changes in environmental conditions or internal physiological states, potentially inducing alterations in the organization of genome duplication as part of these responses.

Given the importance of ensuring the high fidelity duplication of the genetic material, why would the program of replication be so flexible and sensitive to alteration? One possibility is that it may not be particularly important for cells to undergo DNA synthesis in specific ways as long as the genome is fully copied and transmitted. On the other hand, replication domains are a general feature of eukaryotic replication, and cell-type specific conservation of replication timing has been observed in syntenic regions in mouse and human cells [38]. This suggests a functional importance for the organization of DNA replication, an idea that is supported by our earlier work which revealed a role for the replication program in determining the pattern of meiotic recombination in the fission yeast [4]. Furthermore, flexibility in origin usage may promote the coordination of genome duplication with other processes. This may, for instance, act in parallel with mechanisms that prevent and resolve conflicts between the replication and transcription machineries [39,40]. As CDK activity has been shown to drive the waves of gene expression that are associated with different cell cycle phases [29,41], it may be a particularly appropriate input for co-regulating origin selection and transcription during G1/S.

Intriguingly, the three-dimensional organization of the chromosomes in complex eukaryotes is remodeled in early G1, when topologically associating domains (TADs) are established [42,43]. The concomitant organization of the replication timing program as well as the overlap between replication domains and TADs in these systems highlight chromosome conformation as a potential regulator of origin selection. Interestingly, changes in chromosomal organization during the cell cycle have also been reported in the fission yeast [44]. In the context of our study, modulating CDK activity to prolong G1, when large-scale changes in chromosome structure occur, may render particular genomic regions more accessible for assembling replication complexes. This may facilitate a redistribution of replication factors between domains and lead to an equalization of genome-wide origin usage. The overall CDK activity at S phase onset would then represent an additional layer of control that sets the level of efficiencies across the genome. As G1 phase is a sensitive period during which pivotal cell fate decisions are made [45], one appealing suggestion is that the role of CDK in organizing DNA replication may be a vital element in these physiological transitions.

CDK regulation is highly conserved throughout eukaryotes and central to a variety of cellular and developmental processes, including the response to environmental challenges [46]. It is therefore uniquely positioned to integrate the external conditions and intrinsic signals that establish the cellular state, and the capacity of CDK activity to organize genome duplication may be a crucial aspect of its function.

## Materials and methods

### Fission yeast strains and methods

Standard media and methods for fission yeast were used [47,48]. All experiments were carried out in minimal medium plus supplements (EMM6S) at 32°C, except when otherwise noted. The 3-MBPP1 inhibitor (A602960, Toronto Research Chemicals Inc., Canada) was dissolved in DMSO at a stock concentration of 10 mM and added to cultures at the indicated concentrations. The *Schizosaccharomyces pombe* strains used in this study are shown in Table 1.

**Table 1:** Strains used in this study.

Strain	Genotype	Source
DC450	*h+ leu1*∆::*Pcdc13*::*cdc13-L-cdc2as*::*cdc13-3'UTR*::*ura4+* *cdc13*∆::*natMx6 cdc2*∆::*kanMx6 cig1*∆::*hphMX6 cig2*∆::*kanMx6 puc1*∆::*LEU2 ura4-D18*	[30]
JW1045	*h+ leu1*∆::*Pcdc13*::*cdc13-L-cdc2as*::*cdc13-3'UTR*::*ura4+* *cdc13*∆::*natMx6 cdc2*∆::*kanMx6 cig1*∆::*hphMX6 cig2*∆::*kanMx6 puc1*∆::*LEU2 ura4–D18* *ade6-210*::*pFS181_2[Padh1*::*hENT1*::*ade6+] pJL218[Padh1*::*hsvTK*::*his7+]*	This study
JW1241	*h+ leu1*∆::*Pcdc13*::*cdc13-L-cdc2as*::*cdc13-3’UTR*::*ura4+* *cdc13∆*::*natMx6 cdc2∆*::*kanMx6 cig1∆*::*hphMx6 cig2∆*::*kanMx6 puc1∆*::*LEU2 ura4-D18 cdc45-GFP(S65T)-bsd*	This study
PN292	*h+ cdc25-22*	P. Nurse

*cdc2as* = analogue-sensitive mutation, F84G

Synchronization of cells operating with the analog-sensitive fusion protein (Cdc13-Cdc2) was performed as previously described [13]. Specifically, exponentially growing cultures were treated with 1 μM 3-MBPP1 for 2 h 45 min at 32°C, which results in a G2 block. Synchronous entry into the cell cycle was then achieved by removing the inhibitor through filtration of the cultures and 3 successive washes with pre-warmed EMM6S. For the *Control* experiments (Fig 1, S1 Fig), temperature-sensitive *cdc25-22* mutants were grown at permissive temperature (25°C), shifted to restrictive temperature (36.5°C) for 4 h for G2 arrest, and then released to 25°C for re-entry into the cell cycle [32]. Note that the lengths of cell cycle phases, including the duration of S phase, cannot be directly compared between the Cdc13-Cdc2 and *Control* backgrounds as they re-enter the cell cycle after G2 block at very different temperatures (32°C and 25°C, respectively).

For the short G1 delay experiments (G1+5 and G1+15; Figs 2 and 3, S2 and S3 Figs), analog-sensitive Cdc13-Cdc2 cells were synchronized as above and then treated with 2 μM 3-MBPP1 16 min after the initial release from the G2 block, once cells have passed the metaphase to anaphase transition [13]. This treatment arrests cells in G1, preventing S phase onset. The cultures were then released from this G1 block by washing off the inhibitor after 20 min (G1+5) and 30 min (G1+15) of treatment. To estimate the length of the G1 extensions, we considered that in non-G1-arrested cells, mitotic exit has occurred by 15 min after the release from G2 and that S phase starts another 15 min later [13]. Thus, the 20 min treatment with 3-MBPP1 represents a ~5 min extension of G1 (G1+5), while the 30 min condition results in a ~15 min prolongation (G1+15).

To induce a longer delay in G1 (G1+165, Fig 4) as well as to allow cells to enter S phase with different CDK activities (Fig 5), a variation of the above protocol was used. As cells accumulate the Cdc13-Cdc2 fusion protein during a prolonged G1 arrest [13], the cultures initially released from G2 were kept in 2 μM 3-MBPP1 for 30 min and subsequently treated with 20 μM inhibitor. This enabled us to maintain CDK activity below the S phase threshold for much longer periods of time. For our assays, cells were grown in these conditions for another 150 min (165 min extension of G1), during which replication did not occur (Figs 4B and 5A), and then released by filtration in medium containing various concentrations of inhibitor (1, 2.5, 4, or 6 μM). Importantly, it was previously shown that rapid change to inhibitor-free medium after a long G1 block leads to simultaneous entry into S and M phases due to the high level of CDK activity; however, releasing cells into 1 μM 3-MBPP1 prevented this from occurring [13]. Thus, by allowing cells to re-enter the cycle with 1, 2.5, 4, or 6 μM 3-MBPP1 in these conditions, we permitted S phase entry while inhibiting mitosis. Note that in these experiments, cells enter S phase after G1 and therefore undergo a normal round of genome duplication.

### Assessment of DNA content by flow cytometry

For DNA content analysis, cells were fixed in 70% cold ethanol, washed in 50 mM sodium citrate and treated with RNase A (0.1 mg/ml). Samples were then stained using 2 mg/ml propidium iodide, sonicated, and analyzed using a BD Accuri C6 flow cytometer (BD Biosciences, Franklin Lakes, NJ, USA) and the Flowjo analysis software (FlowJo LLC, Ashland, OR, USA). Note that in contrast to a number of model systems, the fission yeast cell cycle displays a short G1 phase, with S phase occurring prior to cytokinesis. Cells therefore have a 2C DNA content during most of their life cycle: in proliferating cells, genome duplication takes place in binucleated cells, giving rise to a transient 4C peak that is then resolved upon cytokinesis. In asynchronous cultures, this 4C peak is barely detectable due to the low percentage of cells undergoing DNA replication at any given time in such populations. Conversely, an elongation of G1 / delay in S phase onset leads to cytokinesis taking place prior to DNA replication and produces a 1C peak. For the flow cytometry profiles in this study, the time points when cells were considered to be undergoing bulk S phase were assessed based on 1) the position of the peak of the major population of cells and 2) the presence of a shoulder to the left of the main peak. For Fig 1B (release from G2 arrest), this shoulder corresponds to cells with DNA contents between 2C and 4C, indicating ongoing DNA synthesis; note that cell division gives rise to 2C cells. For Figs 2B, 4B and 5A (G1 extension), cells with DNA contents between 1C and 2C indicate ongoing DNA synthesis.

### Microarray experiments for determining origin usage

Origin mapping was performed using Agilent 4x44k *S. pombe* arrays (60-mer oligonucleotides every ~250 nucleotides; Agilent Technologies, Santa Clara, CA, USA) as previously described [4]. Competitive hybridization of differentially labeled samples of non-replicating cells vs. cells undergoing DNA synthesis in hydroxyurea (HU; Sigma-Aldrich, St. Louis, MO, USA) was used to determine copy number. The use of HU limits DNA synthesis around the initiation sites, thus permitting the identification of replication origins. Previous studies have validated this method [4,31], which generates origin maps that are comparable to those obtained with other approaches [33,34]. Cells in HU were harvested at a time when bulk S phase would normally be completed in the absence of HU. This was determined for each condition, as shown in Figs 1B, 2B, 4B and 5A.

In the *Control*, 12 mM HU was added when cells were released from the *cdc25-22* G2 arrest, and samples were collected 90 min later. Cells were harvested at the release from G2 for the unreplicated DNA sample. For initial mapping of origins in Cdc13-Cdc2 cells (Fig 1), 12 mM HU was added 10 min after the release from a G2 block, and cells were collected 60 min later (a total of 70 min after the release). For the unreplicated DNA sample, Cdc13-Cdc2 cells were collected 5 min before the release from G2.

For all experiments in which the length of G1 was prolonged (Figs 2–5, S2–S5 Figs), cells were treated with 24 mM HU. Indeed, when 12 mM HU was used in initial G1 extension experiments, we observed broader peaks around initiation sites and the merging of origins; the use of 24 mM HU prevented this further extension of DNA synthesis. As a control for these analyses, we synchronized Cdc13-Cdc2 cells in G2 as above, added 24 mM HU 10 min after the release from G2, and collected samples for origin mapping 60 min after addition of HU (this condition is referred to as G2B; samples for unreplicated DNA were collected 5 min before the release from G2). For the G1+5 and G1+15 conditions, 1) 24 mM HU was added 10 min before the G1 release, 2) samples for unreplicated DNA were collected 5 min later, 3) cells were then released from G1 in the presence of 24 mM HU, and 4) cells were harvested after 60 min. For the G1+165 arrest (identical to the S1 condition), we used a similar protocol as for G1+5 and G1+15 except that cells were released from G1 in the presence of 24 mM HU + 1 μM 3-MBPP1 to prevent mitosis [13]. S phase samples were collected 30 min later. For S2.5, S4, and S6, the same protocol was used with the indicated concentrations of 3-MBPP1. Samples were then collected at the following times after G1 release: 2.5 μM—40 min, 4 μM—45 min, and 6 μM—60 min. These different timings accommodate the changes in S phase length in these experiments (Fig 5A).

To exclude the possibility of a bias in our method due to maintaining cells for different periods of time in HU, we assessed whether the duration of HU exposure has an effect on origin efficiencies. To this end, we collected samples from Cdc13-Cdc2 cells released from G2 arrest and maintained in 24 mM HU for 30 min longer than the time indicated for G2B above (cells were therefore collected 90 min after HU addition; referred to as G2B+30). Our data showed that this does not alter the replication program and that any differences in origin efficiencies between G2B and G2B+30 are not statistically significant (S2 Table, S2C Fig). Our results are consistent with previously published data showing that the length of HU treatment does not alter origin usage profiles [31]. These findings therefore demonstrate that the changes in origin usage that we observe in our study are not due to differences in the duration of HU exposure.

For microarray experiments, genomic DNA was extracted [49] and purified using the Qiagen Genomic DNA kit (Qiagen, Hilden, Germany). Samples were labeled using the BioPrime Plus Array CGH Indirect Genome Labeling Kit (Invitrogen, Carlsbad, CA, USA) with either Alexa 555/647 (Thermo Fisher Scientific, Waltham, MA, USA) or Cy3/Cy5 (GE Healthcare, Little Chalfont, UK) dyes, and 1–2 μg of DNA from the unreplicated and S phase samples were hybridized onto the microarrays. To determine copy number, the geometric means over five consecutive probes were determined throughout the genome for two independent hybridizations of the same samples using a dye-swap, thereby limiting noise and dye bias. The datasets were averaged, and the outliers removed. For each experiment, two biological repeats were performed and averaged. For analysis and comparison of origin efficiencies, the baselines between different conditions, representing unreplicated DNA, were matched and set to 1. For this correction, we surmised that 1) as cells are in HU, there are genomic regions that remain unreplicated in our S phase samples and 2) the unreplicated regions of the genome have the lowest values in each dataset. Thus, for each averaged dataset of biological repeats, we took the lowest 10% of the ratios of replicated/unreplicated DNA, calculated their median, and normalized the dataset to this value. The following correction factors were applied: *Control*, 0.17; Cdc13-Cdc2, 0.13; G2B, 0.1; G2B+30, 0.12; G1+5, 0.18; G1+15, 0.18; G1+165/S1, 0.18; S2.5, 0.2; S4, 0.15; S6, 0.15.

### Origin identification and replication program analyses

S1 Table provides the list of origins and efficiencies in all analyzed conditions. For origin identification in *Control* (*cdc25-22*) and Cdc13-Cdc2 cells (Fig 1, S1 Table), we determined the moving geometric means over five consecutive probes along the chromosomes for each dataset representing the average of two biological repeats. This procedure was then repeated on the resulting datasets for a total of 10 rounds of smoothing. Origins were then identified by the peaks where the copy number ratio of replicated (in HU) vs. unreplicated was greater than 1.05, representing a clear increase over the background after smoothing. Their positions were attributed based on the local maxima followed by visual confirmation. Once the coordinates of the origins were determined, their efficiencies were obtained from the non-smoothed datasets, and a threshold of 1.1 for the copy number was applied. This was further confirmed by visual analysis of the data. When two origins were less than 5 kb apart, they were considered as a single origin and assigned to the position with the higher copy number. This approach is validated by the observation that the extent of replication surrounding an origin in HU can reach 10–15 kb [31]. To compare origin numbers, efficiencies, and positions between two experiments, peaks identified in each dataset were matched in position [4]. Indeed, the precise location of the same origin can vary slightly between experiments, due in part to the resolution of the microarrays and to the extension of replication beyond the initiation sites in these assays. Therefore, when peaks in two different datasets were within a distance of less than 5 kb, they were considered to be the same origin.

For the comparisons of origin efficiencies in Figs 2–5 and S2–S5 Figs, we further refined our origin list. Indeed, as we observed an induction of some inefficient origins in the presented conditions, we visually inspected the averaged and baseline-corrected G1+165/S1 dataset and noted origins with a copy number ≥ 1.1 that were not in our initial list. This resulted in an additional 48 origins that were then added to the list identified in Cdc13-Cdc2 above (S1 Table). For the analyses in Figs 2–5, we therefore used the positions of this larger set of 670 origins and assigned the local maximum values in regions of 5 probes surrounding each origin as their efficiencies.

To determine the regional profiles of origin usage (Figs 1F, 2E, 2F, 3D, 4D, 4E, 5D, 5E, S2D and S5F), the averages of origin efficiencies were determined for continuous windows of ~250 kb (1000 probes) along each chromosome. To evaluate the regional differences in origin usage between different conditions (Figs 2E and 4E, S2D Fig), the differences of the averages in origin efficiencies were determined for continuous windows of ~250 kb (1000 probes).

### Reproducibility of our origin efficiency assays

In addition to the controls described above, we further demonstrated the reproducibility of our methodology for the determination of origin efficiencies and the analysis of differences between the replication program in distinct conditions. First, we established that the individual repeats performed for each condition were highly reproducible (S6 Fig). To this end, we calculated the Spearman’s rank correlation coefficient for each pairwise comparison of repeats and found very strong positive correlations (p-value < 0.001). Complementary to this, we conducted independent samples T-tests (two-sided) and showed no differences between repeats for all of the conditions used in this study (S2 Table) except for S6 (see below). Second, we demonstrated using independent samples T-tests that in contrast to repeat experiments, origin efficiencies in distinct conditions were significantly different (S2 Table). Finally, we determined the regional profiles of origin usage for all of the conditions presented in Figs 2–5 using origin mapping data from individual repeats. These results are displayed in S2D and S5F Figs and reveal virtually identical profiles for both experiments of each condition. We note that a modest difference is observed between the two repeats of S6; this is also suggested by the statistical tests in S2 Table. In this condition, the low level of CDK activity after release from G1 may only be slightly above the S phase threshold, making cells more sensitive to minor experimental variations. All together, we conclude that our methodology for determining origin efficiencies is highly reproducible and robust.

### Chromatin immunoprecipitation and quantitative PCR

Chromatin immunoprecipitation experiments were performed as previously described [35]. Cells were fixed with 1% formaldehyde, lysed in a FastPrep cell disruptor (MP Biomedicals, Santa Ana, CA, USA) and sonicated with a Bioruptor Plus (Diagenode, Seraing, Belgium) to obtain chromatin fragments of ~400–500 bp. The immunoprecipitations (IP) of Cdc45 were carried out overnight at 4°C using an α-GFP antibody (gift from the Nurse lab, diluted 1/2500). Protein G sepharose beads (GE Healthcare, Little Chalfont, UK) or protein G Dynabeads (Thermo Fisher Scientific, Waltham, MA, USA) were then added to the samples and incubated for 4 h at 4°C. IPs were then washed and eluted, and crosslinking was reversed for both IP and Input samples by overnight incubation at 65°C. For quantitative PCR assays, IP and Input DNA were mixed with SYBR Green qPCR Master Mix (Agilent Technologies, Santa Clara, CA, USA) and processed with an ABI 7900 HT (Applied Biosystems, Foster City, CA, USA). A biological repeat was performed for each experiment. Primers used in this study are listed in Table 2.

**Table 2:** Primers used in this study.

Primer	Sequence	Location
OJW63 OJW64	TTGCTTATCTTTTGGGTAGTTTTCG CTTACATTTTCGGGAACTTATTAGTCAA	*ori2004 (oriJW2084)* Chr II: 1545364
AP269 AP270	ATCCAAGTGTAACAAGGTAGGAGT AACGGCCCAAAGCCTTGTAT	*oriJW1072* Chr I: 1315532
AP281 AP282	CTTTTGGCATTTGACGGGGA GCCTACGGATCTTGAATTTGAGG	*oriJW1088* Chr I: 1573759

### ChIP-chip analysis

For Cdc45 ChIP-chip assays, 100 mL of cells were collected at G1/S in the G2B and G1+15 conditions; this was determined as the time when peak Cdc45 binding was observed at early-firing origins (Fig 3A). For G2B, this corresponds to 30 min after release from G2 arrest; for G1+15, cells were collected 3 min after release from the prolonged G1. Note that for these experiments, cells were released into medium without HU. ChIPs were performed as described above using a 1:1000 dilution of the α-GFP antibody and Protein G Dynabeads (Thermo Fisher Scientific, Waltham, MA, USA). For amplification of the ChIP material and labeling for hybridization, the protocol from [50] was used according to [4]. Each ChIP was then hybridized against its reciprocally labeled Input sample (IP/Input). Two biological repeats were performed for each experiment. For all quantitative analyses, probe values were directly used. For visual representation in S3 Fig, the moving geometric means of five consecutive probes were calculated across the genome, and the average of the two experiments were plotted. These profiles show that the sites of Cdc45 binding coincide with origins.

For the analyses of Cdc45 binding in Fig 3B–3D, the Cdc45 levels at origins were determined as follows: for each origin, we took the highest Cdc45 value within a distance of 3 probes of the origin position. To determine the regional profiles of Cdc45 binding (Fig 3C and 3D), we averaged the Cdc45 values assigned to origins over continuous windows of ~250 kb (1000 probes) along each chromosome.

### DNA combing assays

Samples for DNA combing were collected for the S1 and S6 conditions. For these experiments, cells containing the nucleotide transporter (hENT) and thymidine kinase (hsvTK) that allow for incorporation of BrdU into replicating DNA [51] were used (JW1045). Culture conditions were as in Fig 4A, except that 2 μM BrdU (Sigma-Aldrich, St. Louis, MO, USA) was added to the culture medium at the same time as 24 mM HU, 10 min before the release from G1 into the corresponding 3-MBPP1 concentrations. Cells were then collected at the same time as for the microarray experiments, at 30 min and 60 min after the release from a long G1 block for S1 and S6, respectively.

The protocol for the preparation of fibers for DNA combing was as described in [52]. Briefly, cells were treated with 0.1% NaN3 and washed with 50mM EDTA pH = 8 and 0.1% NaN_3_. Cells were then recovered in SP1 (1.2 M D-Sorbitol, 50 mM Citrate phosphate pH 5.6, 40 mM EDTA pH 8) containing 1 μg/μL Zymolyase (AMS Biotechnology, Abingdon, UK) and 0.3 μg/μL Lysing enzymes (Sigma-Aldrich, St. Louis, MO, USA). This mixture was combined with 2% Low Melting Agarose (Bio-Rad, Hercules, CA, USA) in SP1 with 0.2% NaN3, poured into plug molds for pulsed-field gel electrophoresis, and digested at 37°C for 30 min. Agarose plugs were then incubated twice for 30 min at 50°C in DB250X (10 mg/mL N-Lauroylsarcosine sodium salt (Sigma-Aldrich, St. Louis, MO, USA), 1 mg/mL Proteinase K (Roche, Basel, Switzerland), 0.25 M EDTA pH 9.5, 10 mM Tris-HCl pH 9.5), followed by two overnight incubations in DB500X (10 mg/mL N-Lauroylsarcosine sodium salt, 1 mg/mL Proteinase K, 0.5 M EDTA pH 9.5, 10 mM Tris-HCl pH 9.5) at 50°C. They were then washed twice in TE 100X pH 7.5 with 100 mM NaCl, three times in TE 1X pH 7.5, 100 mM NaCl, and once in MES 50 mM pH 5.5 with 100 mM NaCl. Plugs were melted in MES 50 mM pH 5.5 with 100 mM NaCl in Teflon combing receptacles (Genomic Vision, Bagneux, France) at 70°C for 1 h, followed by β-agarase (New England Biolabs, Ipswich, MA, USA) digestion overnight at 42°C.

DNA was combed on silanized coverslips using the Genomic Vision Molecular Combing System (Genomic Vision, Bagneux, France). The immunodetection protocol used was as described in [53]. Fibers were fixed to coverslips at 65°C overnight, dehydrated using ethanol, and denatured in NaOH. Coverslips were incubated in PBST (Phosphate buffered saline, 0.1% Triton X-100) with 1% BSA, followed by sequential incubation with the following antibodies diluted at the indicated concentrations in PBST + 1% BSA: α-BrdU, mouse, IgG1 (1:20; Becton Dickinson, Franklin Lakes, NJ, USA); goat anti-mouse IgG1, Alexa 546 (1:50; Molecular Probes, Eugene, Oregon, USA); α-ssDNA, mouse, IgG2a (1:50; Merck, Darmstadt, Germany); goat anti-mouse IgG2, Alexa 488 (1:50; Molecular Probes, Eugene, Oregon, USA). Coverslips were washed 5 times for 2 min with PBST between antibody incubations. Dako fluorescence mounting medium (Agilent Technologies, Santa Clara, CA, USA) was then added to samples prior to imaging.

Imaging was performed using a 63x objective on an inverted Zeiss Axio Observer (Carl Zeiss AG, Oberkochen, Germany) equipped with a Lumencor Spectra X illumination system (Lumencor Inc., Beaverton, OR, USA). Images were acquired with a Hamamatsu Orca Flash 4.0V2 sCMOS camera (Hamamatsu Photonics, Hamamatsu City, Japan) via the VisiView software (Visitron Systems GmbH, Puchheim, Germany). Fibers were analyzed using Fiji and the Pointpicker plugin. Based on previous studies [54] and our control experiments, a threshold of 0.6 kb (3 pixels) was used as a minimum for positive BrdU staining, and a cutoff of 3 kb (15 pixels) was used to identify stretches of non-BrdU labelled DNA. For S1, we analysed 281 interorigin distances (IODs) in 30 fibers totaling 8351 kb; for S6, we analyzed 242 IODs in 32 fibers totaling 10539 kb (S5D Fig).

### Statistical analyses

Statistics were performed using R Studio. Differences in origin usage between repeats of a given condition and datasets for distinct conditions were analyzed by conducting independent-samples T-tests (two-sided) (S2 Table). These assessments showed that the alterations in origin usage observed upon modulating CDK timing and activity are statistically significant, while this is not the case for individual repeats of a given condition. The Spearman’s rank correlation coefficient was evaluated for the comparisons in Figs 1F and 2E, Figs 3B, 3D and S6. For DNA combing analysis, differences in interorigin distances between conditions were evaluated by conducting independent-samples T-tests (two-sided) (S5D Fig).

### Accession number

The array data reported in this paper have been deposited in the NIH GEO database and has been assigned the accession number GSE88714.

## Supporting information

S1 Fig Replication origin usage in *Control* and Cdc13-Cdc2 cells. **AI-III)** Detailed view of the origin usage profiles of *Control* (black) and Cdc13-Cdc2 (red) cells as in Fig 1C. x-axis: chromosome coordinates, y-axis: origin efficiencies. I, II, and III each display one of the three chromosomes of fission yeast. **B)** Origin usage characteristics in the *Control* and Cdc13-Cdc2 backgrounds. Note that although the average origin efficiency in *Control* cells is slightly higher than that in Cdc13-Cdc2, S phase appears to be longer. This is due to the experimental differences in the growth conditions of the cells: the *Control* undergoes S phase at 25°C, while Cdc13-Cdc2 is maintained at 32°C.
(PDF)

S2 Fig Replication origin usage in cells with a prolonged G1 phase. **AI-III)** Detailed view of the origin usage profiles of G2B (black), G1+15 (blue), and G1+5 (red) as in Fig 2C. x-axis: chromosome coordinates, y-axis: origin efficiencies. **B)** Pairwise comparisons of origin efficiencies. Left panel: x-axis: efficiencies in G2B, y-axis: efficiencies in G1+15; right panel: x-axis: efficiencies in G2B, y-axis: efficiencies in G1+5. Each dot represents an origin. The dashed lines represent efficiencies if they were identical in the two compared backgrounds. **C)** Analysis of origin efficiencies in cells that have undergone different lengths of HU treatment. Cdc13-Cdc2 cells were synchronized as in Fig 1A but treated with 24 mM HU for either 60 min (G2B) or 90 min (G2B+30) (specifically, cells were harvested 70 min and 100 min after release from G2, respectively). G2B data are as in S2B Fig. Origin efficiencies in the two conditions are virtually identical despite significant differences in the duration of the HU treatment (see also S2 Table). x-axis: efficiencies in G2B; y-axis: efficiencies in G2B+30. Each dot represents an origin. The dashed line represents efficiencies if they were identical in the two compared backgrounds. **D)** Regional analysis of origin efficiencies in individual replicate experiments in the G2B (Top), G1+5 (Middle), and G1+15 (Bottom) conditions. Average origin efficiencies were determined in continuous windows of 1000 probes (~250 kb). Black: experiment 1, red: experiment 2. Dashed line indicates the mean efficiency of all origins in each experiment; the corresponding experiment is indicated by the color. The average efficiency values are as follows: G2B: 25.4% and 24.5%; G1+5: 33.0% and 30.8%; G1+15: 29.4% and 29.5. x-axis: chromosome coordinates, y-axis: average origin efficiencies. These results show the high level of reproducibility of our datasets.
(PDF)

S3 Fig Genome-wide alterations in Cdc45 binding following a short G1 extension. **A)** Profiles of origin efficiencies (black, top) vs. Cdc45 binding (red, bottom) for G2B. x-axis: chromosome coordinates; top y-axis: origin efficiency; bottom y-axis: Cdc45 level (IP/input). Origin efficiency data are as in Fig 2C and S2A Fig. **B)** Detailed views of origin efficiencies (black, right y-axis) and Cdc45 binding (red, left y-axis) in representative regions of the genome for G2B. x-axis: chromosome coordiates. **C)** Profiles of origin efficiencies (black, top) vs. Cdc45 binding (red, bottom) for G1+15. x-axis: chromosome coordinates; top y-axis: origin efficiency; bottom y-axis: Cdc45 level (IP/input). Origin efficiency data are as in Fig 2C and S2A Fig. **D)** Detailed views of origin efficiencies (black, right y-axis) and Cdc45 binding (red, left y-axis) in representative regions of the genome for G1+15. x-axis: chromosome coordinates. **EI-III)** Detailed views of Cdc45 binding in G2B (black, top panels, as in *A*) and G1+15 (red, bottom panels, as in *C*). x-axis: chromosome coordinates; y-axis: Cdc45 level (IP/input). **F)** Histograms displaying the deviations of Cdc45 binding and origin efficiency from their genome-wide averages in G2B and G1+15. For each parameter, the average of the regional profile was calculated, and the deviation of each point in the regional profile from the corresponding mean was determined and plotted. The bimodal distributions in G2B represent efficient and inefficient regions; this is not observed for G1+15, where efficiencies between regions are more similar. This analysis shows a clear equalization of both Cdc45 and origin efficiencies in G1+15 compared to G2B.
(PDF)

S4 Fig Comparison of replication origin usage in short and long G1 extensions. **AI-III)** Detailed view of the origin usage profiles of G2B (black), G1+15 (blue), and G1+165 (green) as in Fig 4C. x-axis: chromosome coordinates, y-axis: origin efficiencies. **B)** Pairwise comparisons of origin efficiencies in the different G1 extensions. Left panel: x-axis: efficiencies in G2B, y-axis: efficiencies in G1+165; right panel: x-axis: efficiencies in G+15, y-axis: efficiencies in G1+165. Each dot represents an origin. The dashed black lines represent efficiencies if they were identical in the two compared backgrounds. **C)** Origin usage characteristics in the G2B and G1 extension conditions. **D)** Time courses of chromatin immunoprecipitation of Cdc45 in G1+165. Note that these experiments were performed as in Fig 4A but without the addition of HU to allow progression through S phase. Efficiencies of the origins analyzed in the G1+165 condition are as follows: *ori2084* (*ori2004*): 55%, *oriJW1072*: 33%, *oriJW1088*: 50%. x-axis: time after release from G1 arrest; y-axis: Cdc45 binding (% IP). *n* = 2, a representative experiment is displayed.
(PDF)

S5 Fig Replication origin usage in cells that enter S phase with different levels of CDK activity. **AI-III)** Detailed view of the origin usage profiles of S1 (black), S2.5 (red), S4 (blue), and S6 (purple) as in Fig 5B. x-axis: chromosome coordinates, y-axis: origin efficiencies. **B)** Pairwise comparisons of origin efficiencies in cells entering S phase with different levels of CDK activity. The dashed line represents efficiencies if they were identical in two compared backgrounds. Black dots: S2.5 vs. S1; red dots: S4 vs. S1; blue dots: S6 vs. S1. x- and y-axes: origin efficiencies in the indicated conditions. Each dot represents an origin. **C)** Origin usage characteristics for S1, S2.5, S4, and S6. **D)** DNA combing analysis of interorigin distances (IOD) in the S1 and S6 conditions. For S1, 281 IODs were analyzed in 30 independent fibers totaling 8351 kb. For S6, 242 IODs were analyzed in 32 independent fibers totaling 10539 kb. Box and whiskers plot, with outliers represented by circles. For ease of visualization, IODs > 100 kb were not displayed in the graph. This represents only 3 points in S1 (out of 281) and 7 points in S6 (out of 242). S1 has a median IOD of 15.2 kb, and S6 has a median IOD of 17.8 kb. This is consistent with a decrease in origin usage in S6 (average efficiency = 23.4%) compared to S1 (average efficiency = 33.4%). Independent-samples T-tests (two-sided) showed that these IOD values were significantly different (**: p-value = 0.0029). These results support the overall decrease in origin usage observed in our population, genome-wide analyses when cells enter S phase with lower levels of CDK activity. **E)** Pairwise comparisons of origin efficiencies in S2.5 vs. G1+15. The dashed black line represents efficiencies if they were identical in the two compared backgrounds. x- and y-axes: efficiencies in the indicated conditions. Each dot represents an origin. **F)** Regional analysis of origin efficiencies in individual replicate experiments in the S1, S2.5, S4, and S6 conditions. Average origin efficiencies were determined in continuous windows of 1000 probes (~250 kb). Black: experiment 1, red: experiment 2. Dashed line indicates the mean efficiency of all origins in each experiment; the corresponding experiment is indicated by the color. Dotted lines mark 20% and 40% efficiency. The average efficiency values are as follows: S1: 33.3% and 33.6%; S2.5: 30.0% and 29.7%; S4: 25.1% and 25.4%; S6: 24.9% and 21.9%. x-axis: chromosome coordinates, y-axis: average origin efficiencies. Note that the repeats of S6 are detectably different (see also S2 Table). Indeed, the low level of CDK activity in cells just after release from G1 in this condition may be just above the threshold for S phase entry, which may render them more sensitive to small variations in experimental conditions.
(PDF)

S6 Fig Comparisons of origin efficiencies from individual experiments for each condition. **A-J)** Graphs comparing the two repeats of origin mapping experiments performed in all conditions in this study. The Spearmans’ rank correlation coefficient (ρ) for each comparison is displayed. ***: p < 0.001. The dashed black lines represent efficiencies if they were identical in the two repeats. x- and y-axes: origin efficiencies in the indicated conditions. These results show the high level of reproducibility of our datasets.
(PDF)

S1 Table List of origins identified in all experimental conditions along with their efficiencies. (XLSX)

S2 Table Statistical analyses of the differences in origin usage between conditions. Independent-samples T-tests (two-sided) were applied. Statistically significant differences are shown in red.
(DOCX)
