# Supplementary material for: CDK activity provides temporal and quantitative cues for organizing genome duplication
Source: PLoS Genet. 2018 Feb 21;14(2):e1007214. doi: 10.1371/journal.pgen.1007214 (PMC5821308; doi:10.1371/journal.pgen.1007214)
Supplement: S1 Fig — AI-III) Detailed view of the origin usage profiles of Control (black) and Cdc13-Cdc2 (red) cells as in Fig 1C. x-axis: chromosome coordinates, y-axis: origin efficiencies. I, II, and III each display one of the three chromosomes of fission yeast. B) Origin usage characteristics in the Control and Cdc13-Cdc2 backgrounds. Note that although the average origin efficiency in Control cells is slightly higher than that in Cdc13-Cdc2, S phase appears to be longer. This is due to the experimental differences in the growth conditions of the cells: the Control undergoes S phase at 25°C, while Cdc13-Cdc2 is maintained at 32°C. (PDF) [file pgen.1007214.s001.pdf]

**Figure S1**

**A_I**

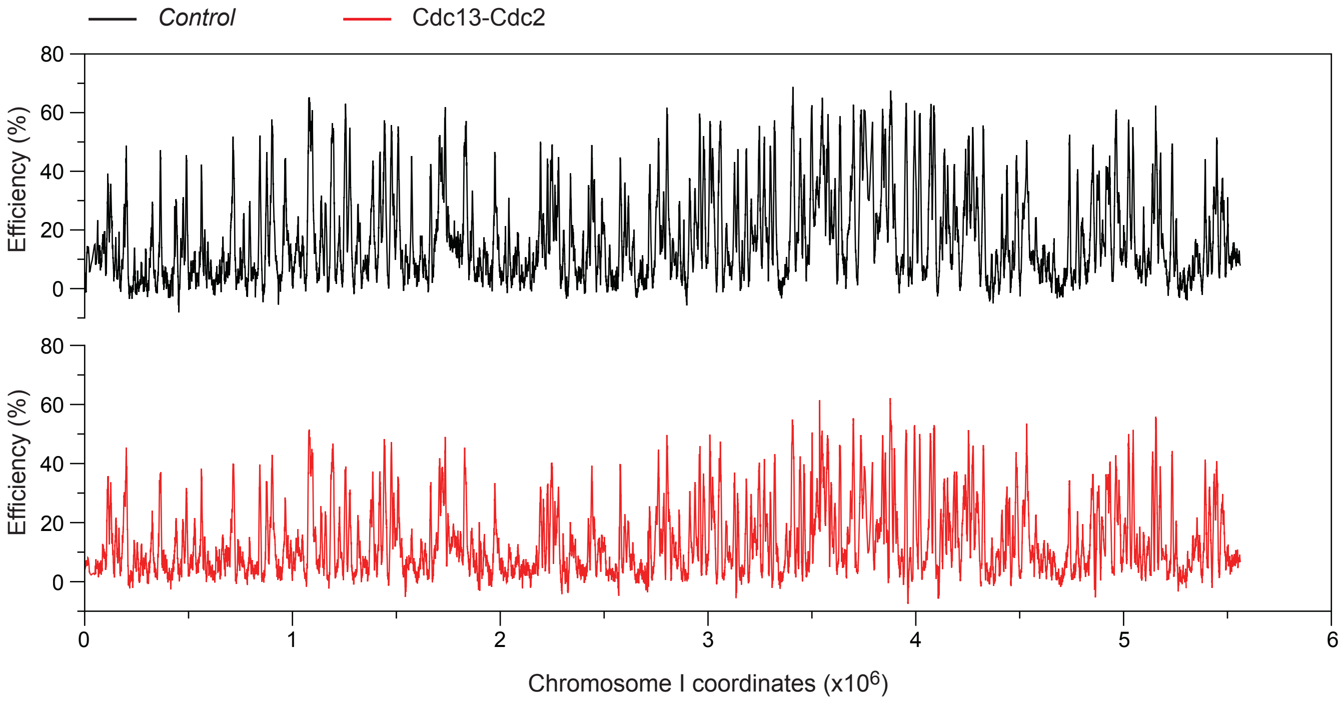


**A_II**

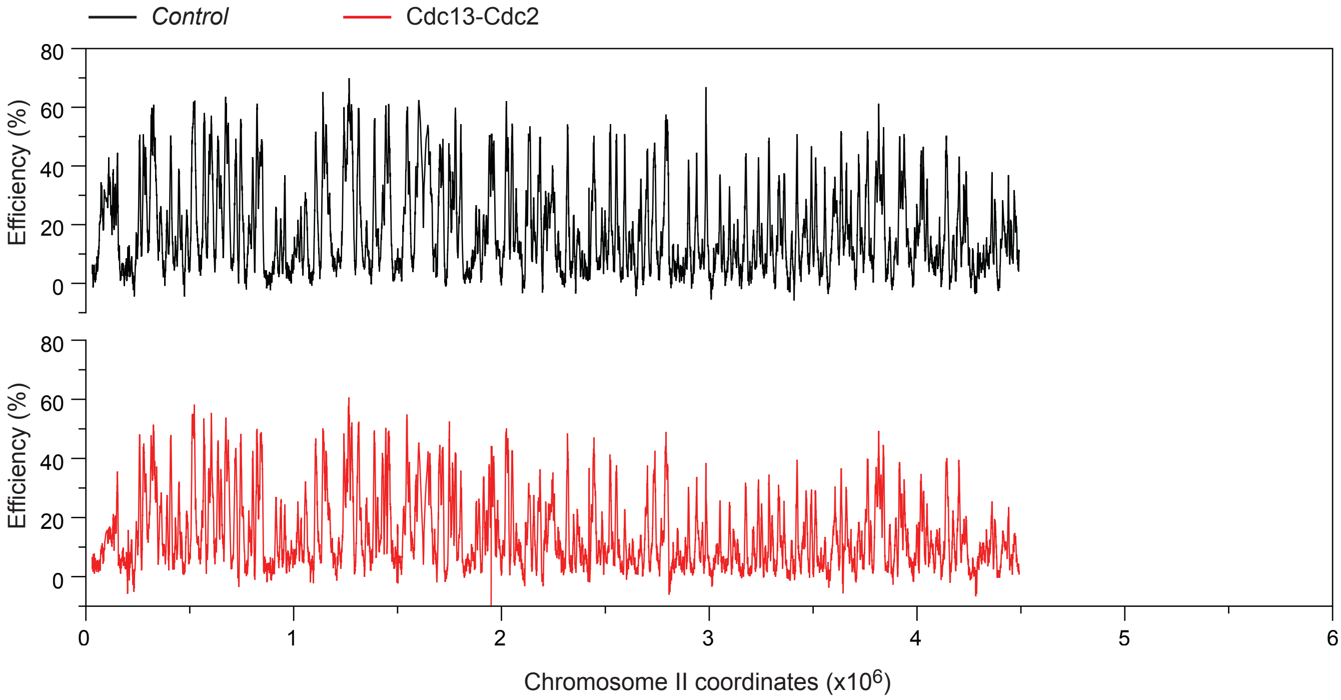

**Figure S1**

**A_III**

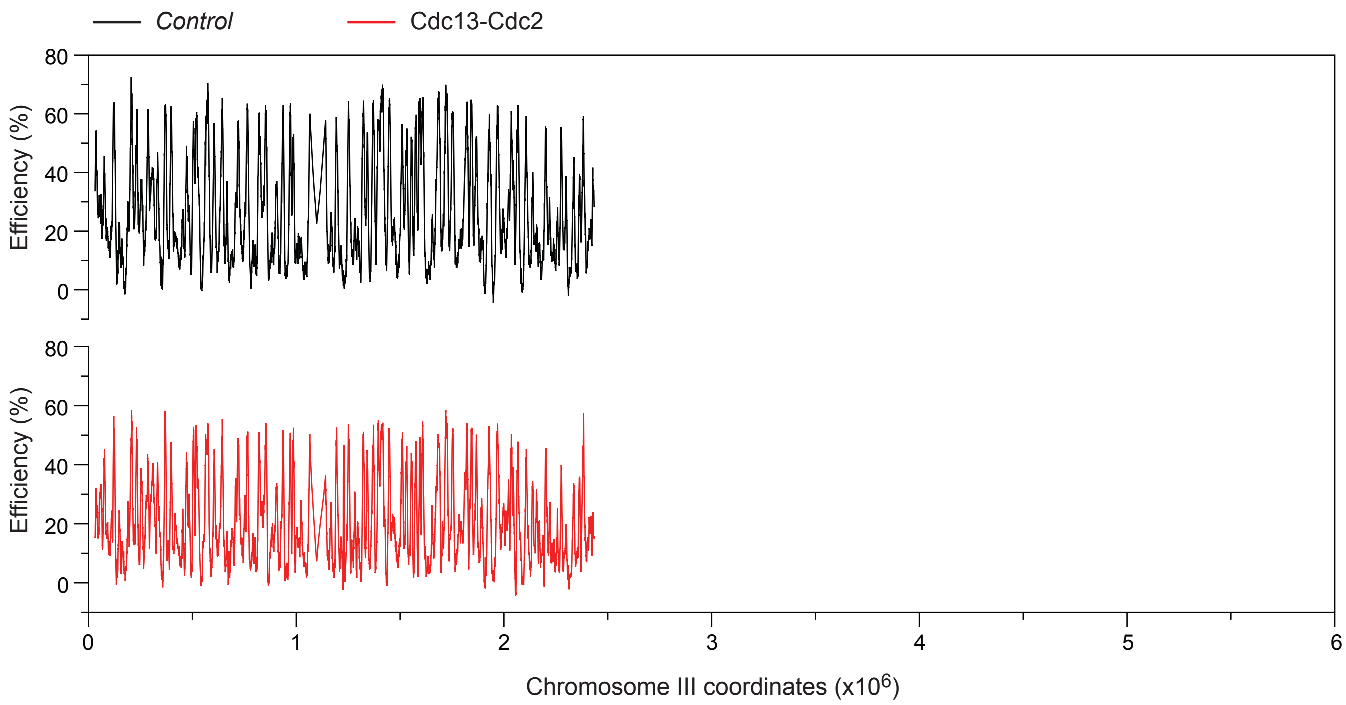


**B**

| | *Control* | Cdc13-Cdc2 |
|---|---|---|
| Total number of origins | 660 | 622 |
| Average origin efficiency (%) | 33.3 | 29.1 |
| Average efficiency difference for all origins (%) | 4.2 | |
| Number of origins in common | 598 | |
| % in common | 91 | 96 |
| Number of origins unique to each condition | 63 | 24 |
| % unique | 9.4 | 3.9 |
| Average efficiency of unique origins (%) | 15.7 | 14.0 |
| Standard deviation of efficiencies of unique origins | 5.8 | 4.5 |

**Fig S1. Replication origin usage in *Control* and Cdc13-Cdc2 cells. AI-III)** Detailed view of the origin usage profiles of *Control* (black) and Cdc13-Cdc2 (red) cells as in Fig 1C. x-axis: chromosome coordinates, y-axis: origin efficiencies. I, II, and III each display one of the three chromosomes of fission yeast. **B)** Origin usage characteristics in the *Control* and Cdc13-Cdc2 backgrounds. Note that although the average origin efficiency in *Control* cells is slightly higher than that in Cdc13-Cdc2, S phase appears to be longer. This is due to the experimental differences in the growth conditions of the cells: the *Control* undergoes S phase at 25 ºC, while Cdc13-Cdc2 is maintained at 32 ºC.
