# Supplementary material for: CDK activity provides temporal and quantitative cues for organizing genome duplication
Source: PLoS Genet. 2018 Feb 21;14(2):e1007214. doi: 10.1371/journal.pgen.1007214 (PMC5821308; doi:10.1371/journal.pgen.1007214)
Supplement: S2 Fig — AI-III) Detailed view of the origin usage profiles of G2B (black), G1+15 (blue), and G1+5 (red) as in Fig 2C. x-axis: chromosome coordinates, y-axis: origin efficiencies. B) Pairwise comparisons of origin efficiencies. Left panel: x-axis: efficiencies in G2B, y-axis: efficiencies in G1+15; right panel: x-axis: efficiencies in G2B, y-axis: efficiencies in G1+5. Each dot represents an origin. The dashed lines represent efficiencies if they were identical in the two compared backgrounds. C) Analysis of origin efficiencies in cells that have undergone different lengths of HU treatment. Cdc13-Cdc2 cells were synchronized as in Fig 1A but treated with 24 mM HU for either 60 min (G2B) or 90 min (G2B+30) (specifically, cells were harvested 70 min and 100 min after release from G2, respectively). G2B data are as in S2B Fig. Origin efficiencies in the two conditions are virtually identical despite significant differences in the duration of the HU treatment (see also S2 Table). x-axis: efficiencies in G2B; y-axis: efficiencies in G2B+30. Each dot represents an origin. The dashed line represents efficiencies if they were identical in the two compared backgrounds. D) Regional analysis of origin efficiencies in individual replicate experiments in the G2B (Top), G1+5 (Middle), and G1+15 (Bottom) conditions. Average origin efficiencies were determined in continuous windows of 1000 probes (~250 kb). Black: experiment 1, red: experiment 2. Dashed line indicates the mean efficiency of all origins in each experiment; the corresponding experiment is indicated by the color. The average efficiency values are as follows: G2B: 25.4% and 24.5%; G1+5: 33.0% and 30.8%; G1+15: 29.4% and 29.5. x-axis: chromosome coordinates, y-axis: average origin efficiencies. These results show the high level of reproducibility of our datasets. (PDF) [file pgen.1007214.s002.pdf]

Figure S2

A_I

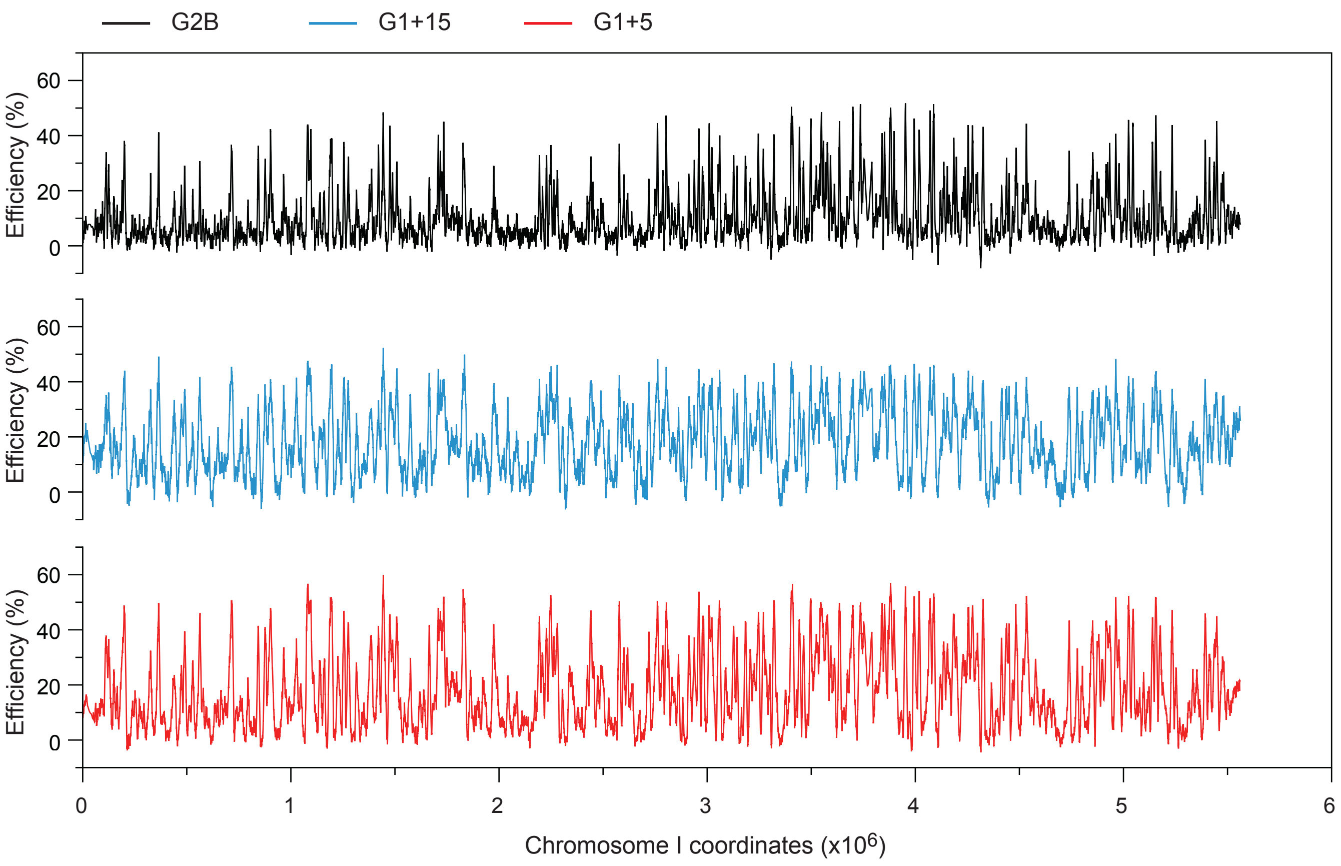

A_II

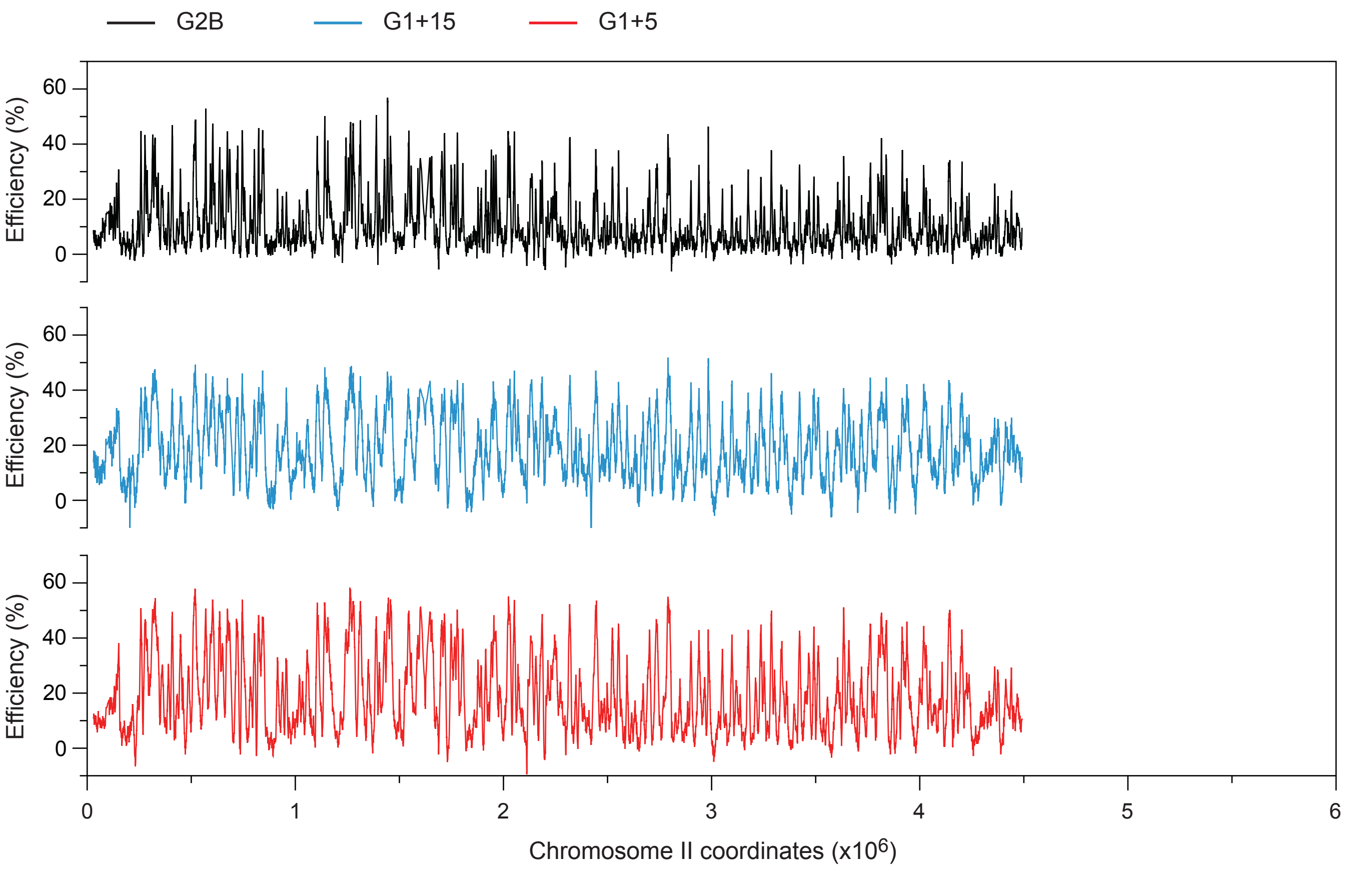

Figure S2

A_III

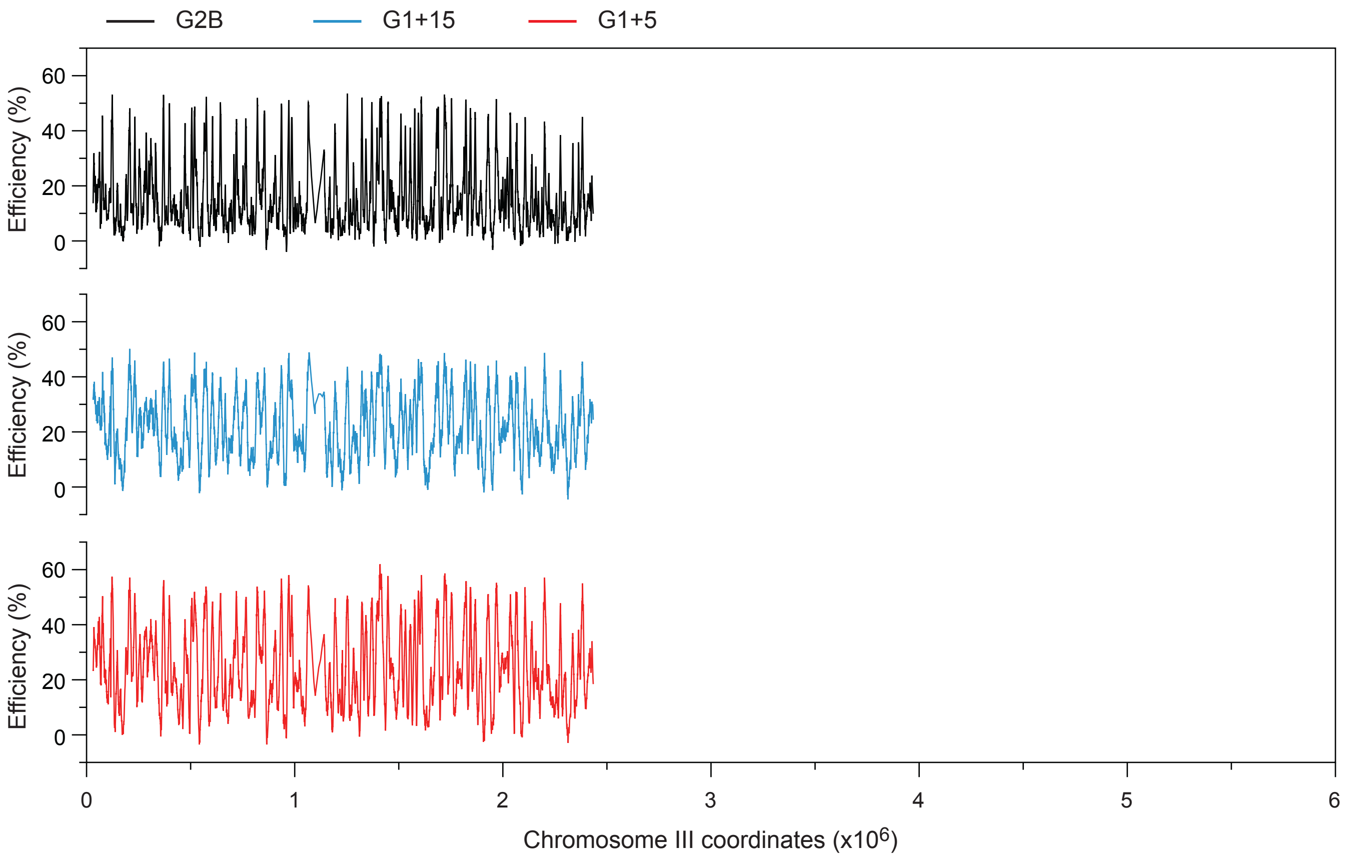

B

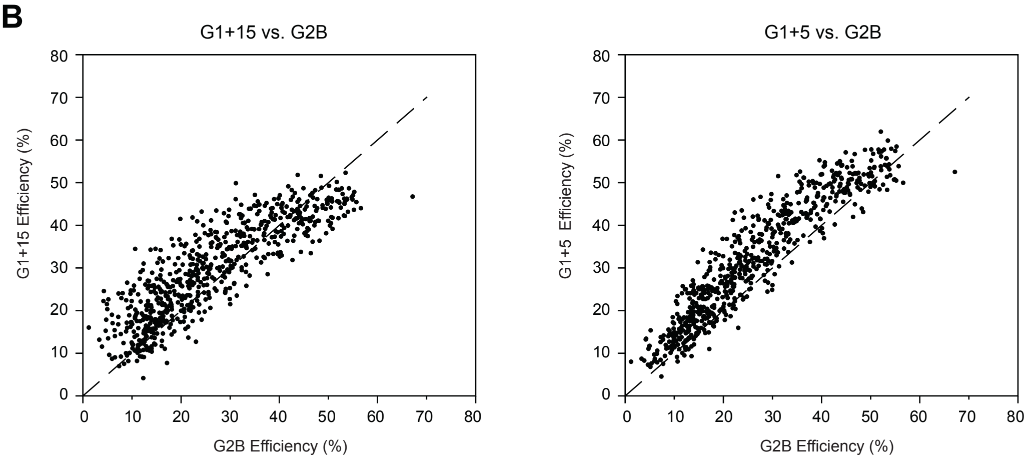

C

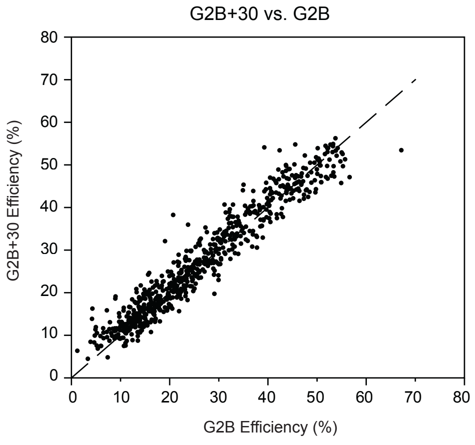

Figure S2

D

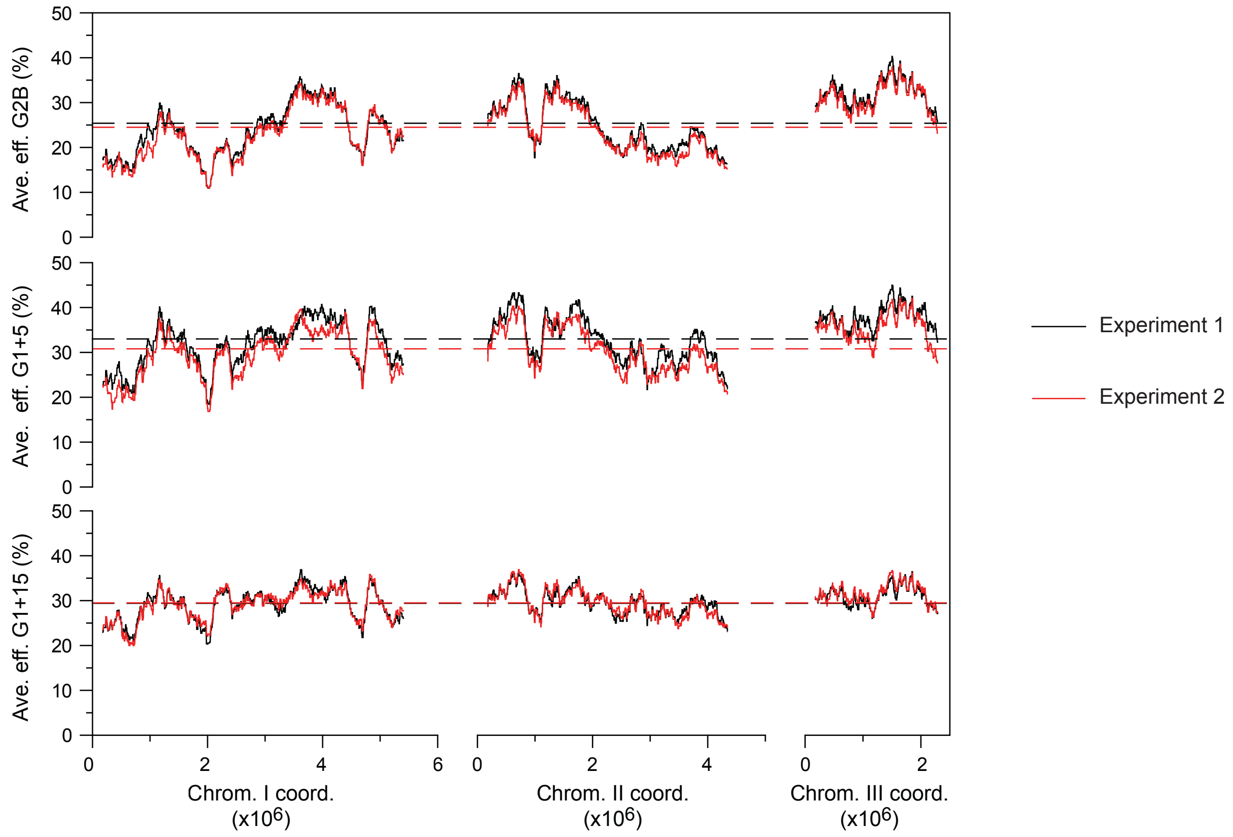

Ave. eff. G2B (%)
Ave. eff. G1+5 (%)
Ave. eff. G1+15 (%)
0
10
20
30
40
50
0
2
4
6
Chrom. I coord. (x10^6)
0
2
4
Chrom. II coord. (x10^6)
0
2
Chrom. III coord. (x10^6)
Experiment 1
Experiment 2

Fig S2. Replication origin usage in cells with a prolonged G1 phase. AI-III) Detailed view of the origin usage profiles of G2B (black), G1+15 (blue), and G1+5 (red) as in Fig 2C. x-axis: chromosome coordinates, y-axis: origin efficiencies. **B)** Pairwise comparisons of origin efficiencies. Left panel: x-axis: efficiencies in G2B, y-axis: efficiencies in G1+15; right panel: x-axis: efficiencies in G2B, y-axis: efficiencies in G1+5. Each dot represents an origin. The dashed lines represent efficiencies if they were identical in the two compared backgrounds. **C)** Analysis of origin efficiencies in cells that have undergone different lengths of HU treatment. Cdc13-Cdc2 cells were synchronized as in Fig 1A but treated with 24 mM HU for either 60 min (G2B) or 90 min (G2B+30) (specifically, cells were harvested 70 min and 100 min after release from G2, respectively). G2B data are as in Fig S2B. Origin efficiencies in the two conditions are virtually identical despite significant differences in the duration of the HU treatment (see also Table S2). x-axis: efficiencies in G2B; y-axis: efficiencies in G2B+30. Each dot represents an origin. The dashed line represents efficiencies if they were identical in the two compared backgrounds. **D)** Regional analysis of origin efficiencies in individual replicate experiments in the G2B (Top), G1+5 (Middle), and G1+15 (Bottom) conditions. Average origin efficiencies were determined in continuous windows of 1000 probes (~250 kb). Black: experiment 1, red: experiment 2. Dashed line indicates the mean efficiency of all origins in each experiment; the corresponding experiment is indicated by the color. The average efficiency values are as follows: G2B: 25.4% and 24.5%; G1+5: 33.0% and 30.8%; G1+15: 29.4% and 29.5. x-axis: chromosome coordinates, y-axis: average origin efficiencies. These results show the high level of reproducibility of our datasets.
