# Supplementary material for: CDK activity provides temporal and quantitative cues for organizing genome duplication
Source: PLoS Genet. 2018 Feb 21;14(2):e1007214. doi: 10.1371/journal.pgen.1007214 (PMC5821308; doi:10.1371/journal.pgen.1007214)
Supplement: S3 Fig — A) Profiles of origin efficiencies (black, top) vs. Cdc45 binding (red, bottom) for G2B. x-axis: chromosome coordinates; top y-axis: origin efficiency; bottom y-axis: Cdc45 level (IP/input). Origin efficiency data are as in Fig 2C and S2A Fig. B) Detailed views of origin efficiencies (black, right y-axis) and Cdc45 binding (red, left y-axis) in representative regions of the genome for G2B. x-axis: chromosome coordiates. C) Profiles of origin efficiencies (black, top) vs. Cdc45 binding (red, bottom) for G1+15. x-axis: chromosome coordinates; top y-axis: origin efficiency; bottom y-axis: Cdc45 level (IP/input). Origin efficiency data are as in Fig 2C and S2A Fig. D) Detailed views of origin efficiencies (black, right y-axis) and Cdc45 binding (red, left y-axis) in representative regions of the genome for G1+15. x-axis: chromosome coordinates. EI-III) Detailed views of Cdc45 binding in G2B (black, top panels, as in A) and G1+15 (red, bottom panels, as in C). x-axis: chromosome coordinates; y-axis: Cdc45 level (IP/input). F) Histograms displaying the deviations of Cdc45 binding and origin efficiency from their genome-wide averages in G2B and G1+15. For each parameter, the average of the regional profile was calculated, and the deviation of each point in the regional profile from the corresponding mean was determined and plotted. The bimodal distributions in G2B represent efficient and inefficient regions; this is not observed for G1+15, where efficiencies between regions are more similar. This analysis shows a clear equalization of both Cdc45 and origin efficiencies in G1+15 compared to G2B. (PDF) [file pgen.1007214.s003.pdf]

Figure S3

A

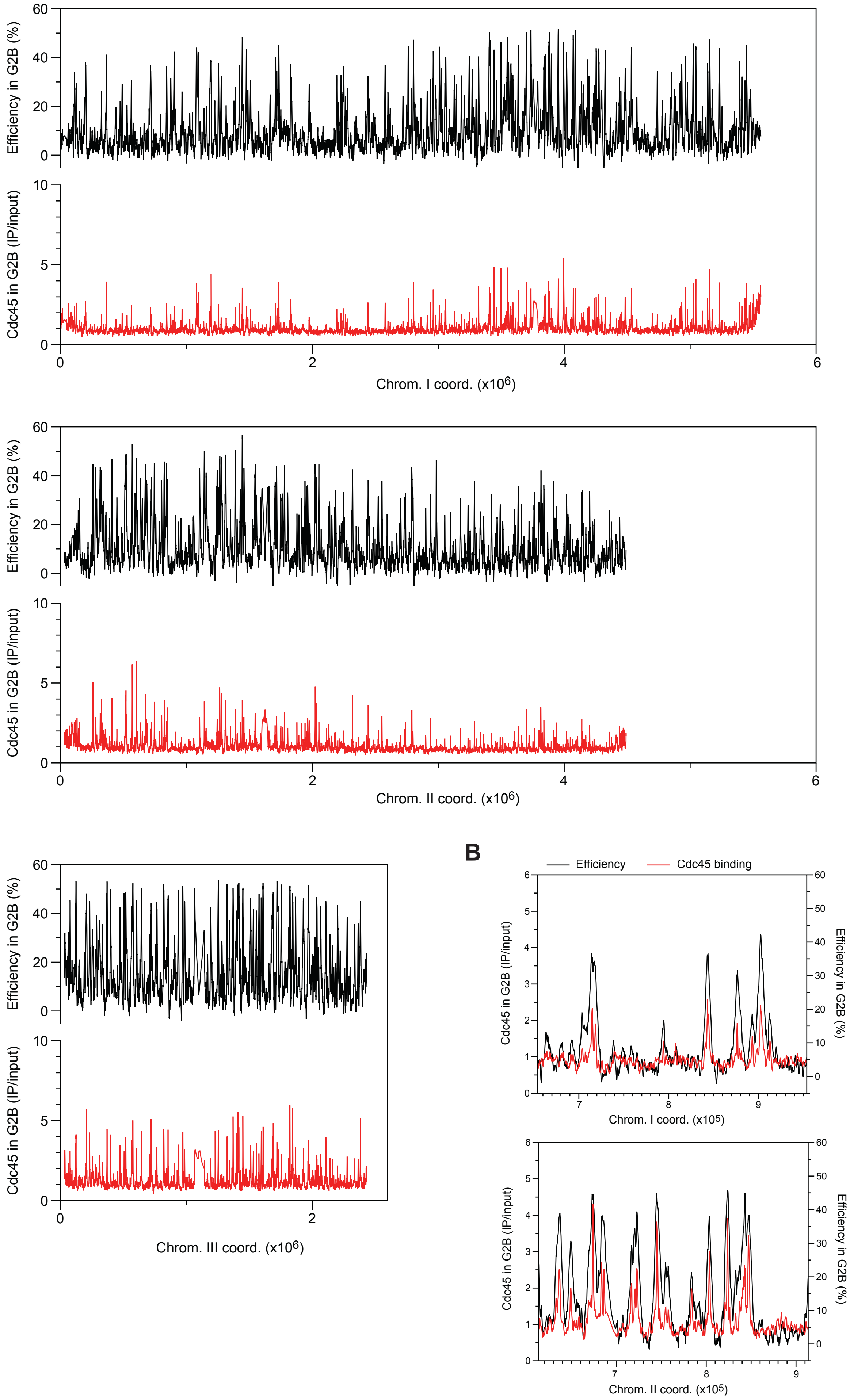

Efficiency in G2B (%)
Cdc45 in G2B (IP/input)
Chrom. I coord. (x10^6)
Chrom. II coord. (x10^6)
Chrom. III coord. (x10^6)
B
Efficiency
Cdc45 binding
Cdc45 in G2B (IP/input)
Efficiency in G2B (%)
Chrom. I coord. (x10^5)
Chrom. II coord. (x10^5)

Figure S3

C

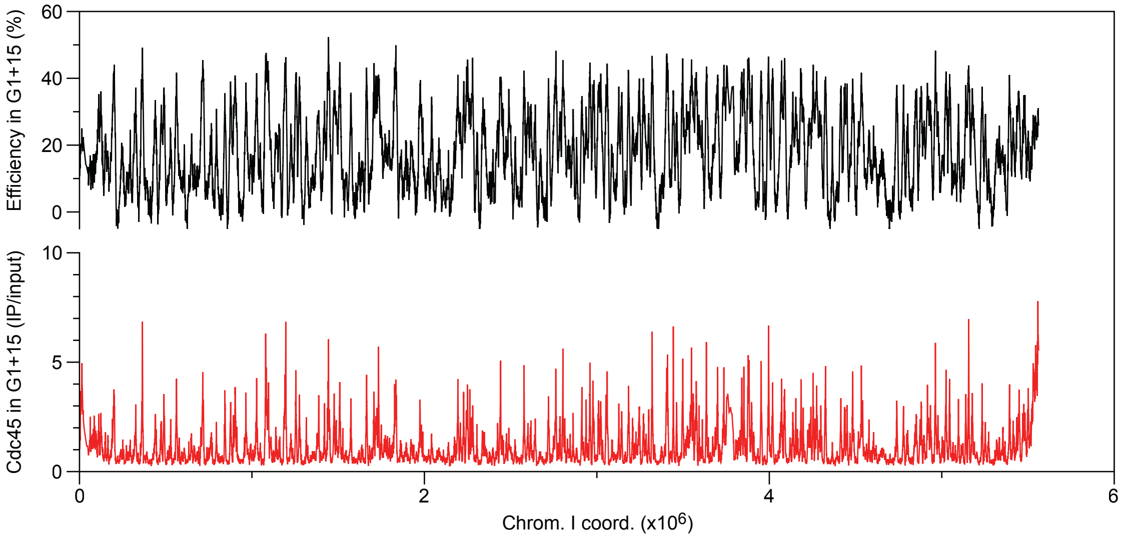
Efficiency in G1+15 (%)
Cdc45 in G1+15 (IP/input)
Chrom. I coord. (x10^6)

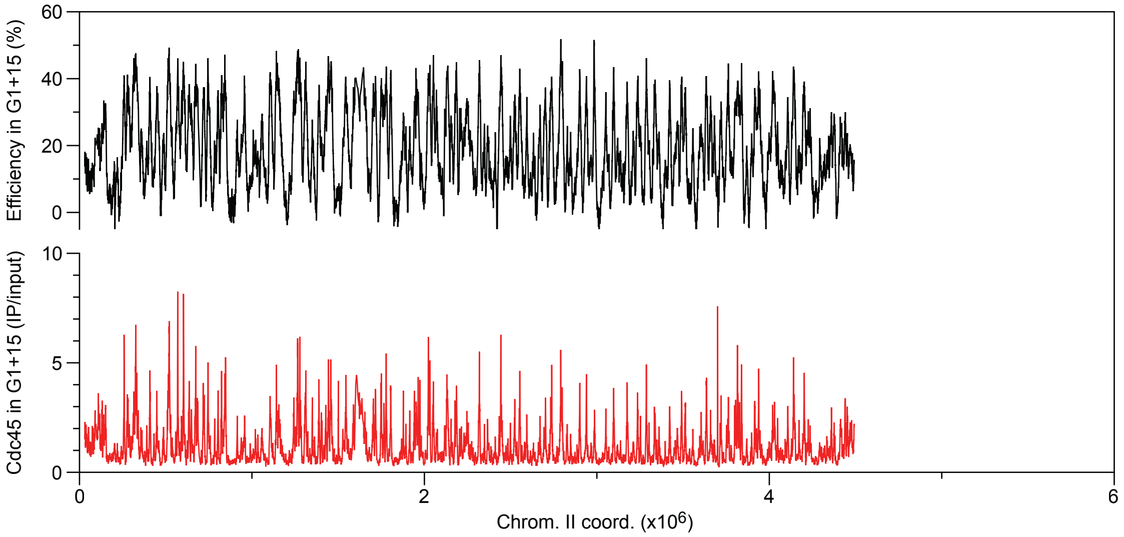
Efficiency in G1+15 (%)
Cdc45 in G1+15 (IP/input)
Chrom. II coord. (x10^6)

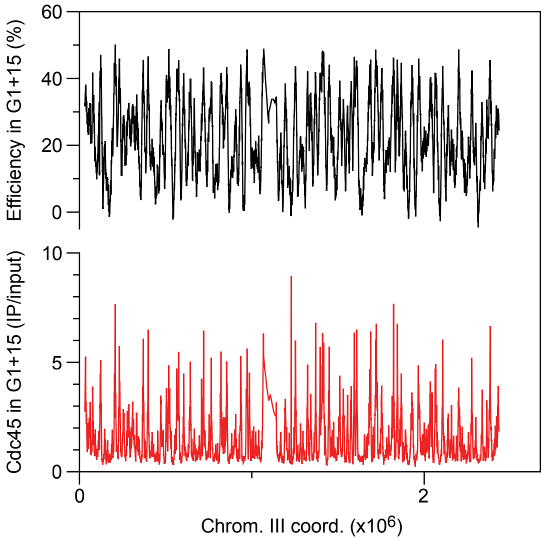
Efficiency in G1+15 (%)
Cdc45 in G1+15 (IP/input)
Chrom. III coord. (x10^6)

D

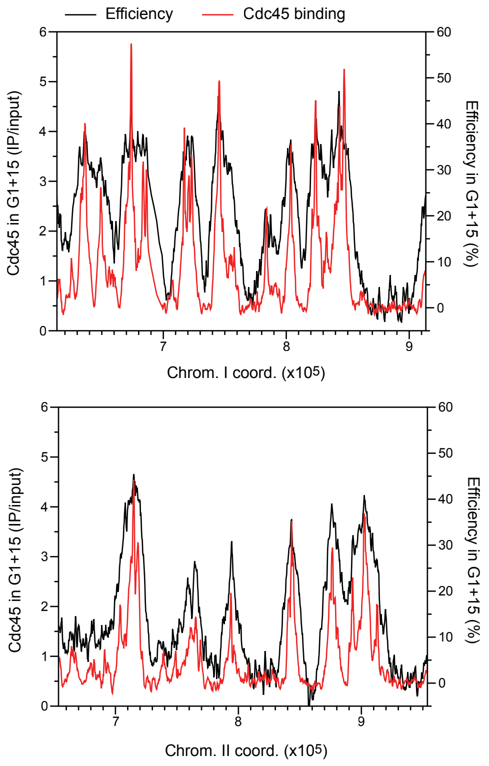
Efficiency
Cdc45 binding
Cdc45 in G1+15 (IP/input)
Efficiency in G1+15 (%)
Chrom. I coord. (x10^5)
Cdc45 in G1+15 (IP/input)
Efficiency in G1+15 (%)
Chrom. II coord. (x10^5)

Figure S3

E_I

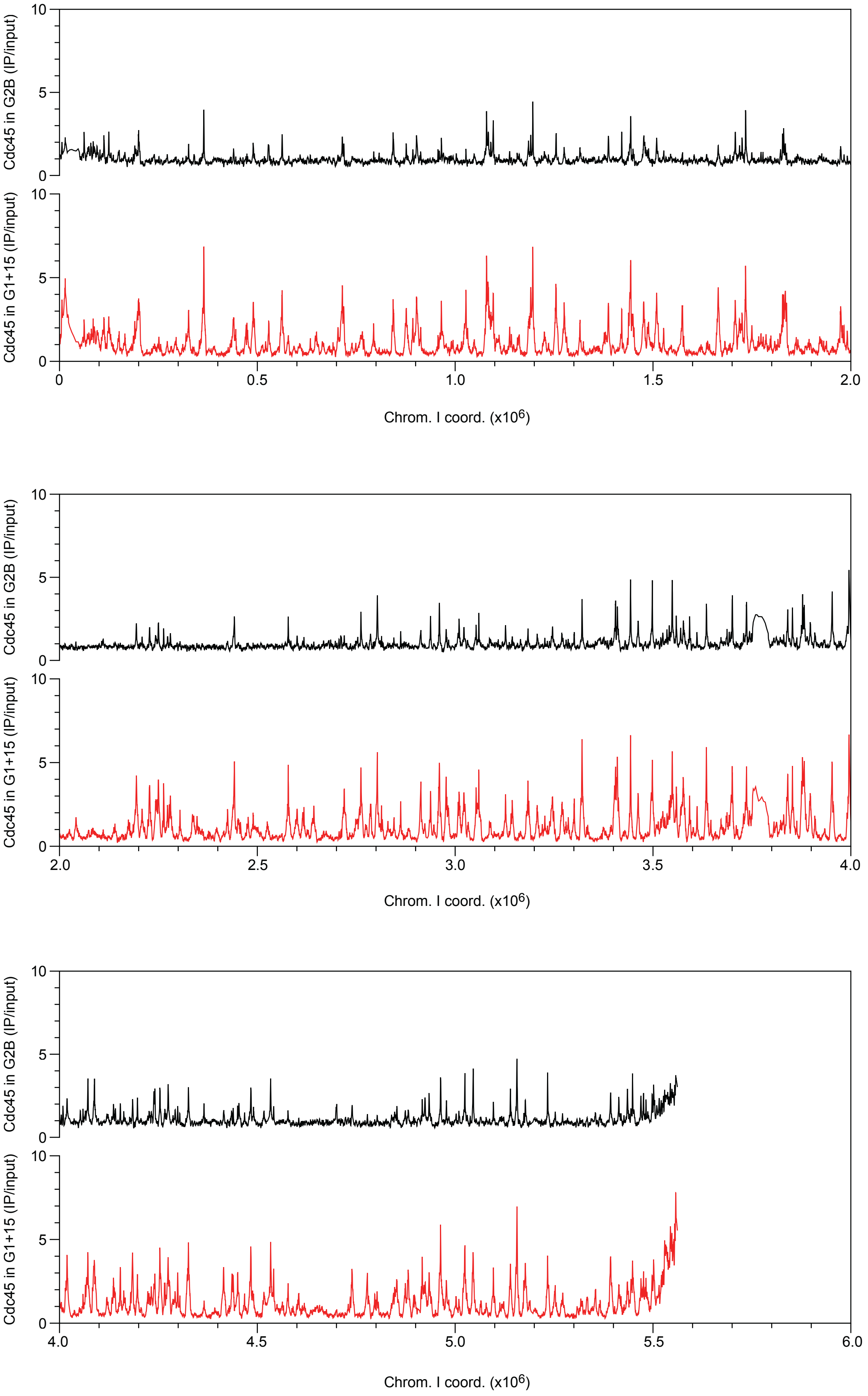

Cdc45 in G2B (IP/input)
Cdc45 in G1+15 (IP/input)
10
5
0
0
0.5
1.0
1.5
2.0
Chrom. I coord. (x10^6)
Cdc45 in G2B (IP/input)
Cdc45 in G1+15 (IP/input)
2.0
2.5
3.0
3.5
4.0
Chrom. I coord. (x10^6)
Cdc45 in G2B (IP/input)
Cdc45 in G1+15 (IP/input)
4.0
4.5
5.0
5.5
6.0
Chrom. I coord. (x10^6)

Figure S3

E_II

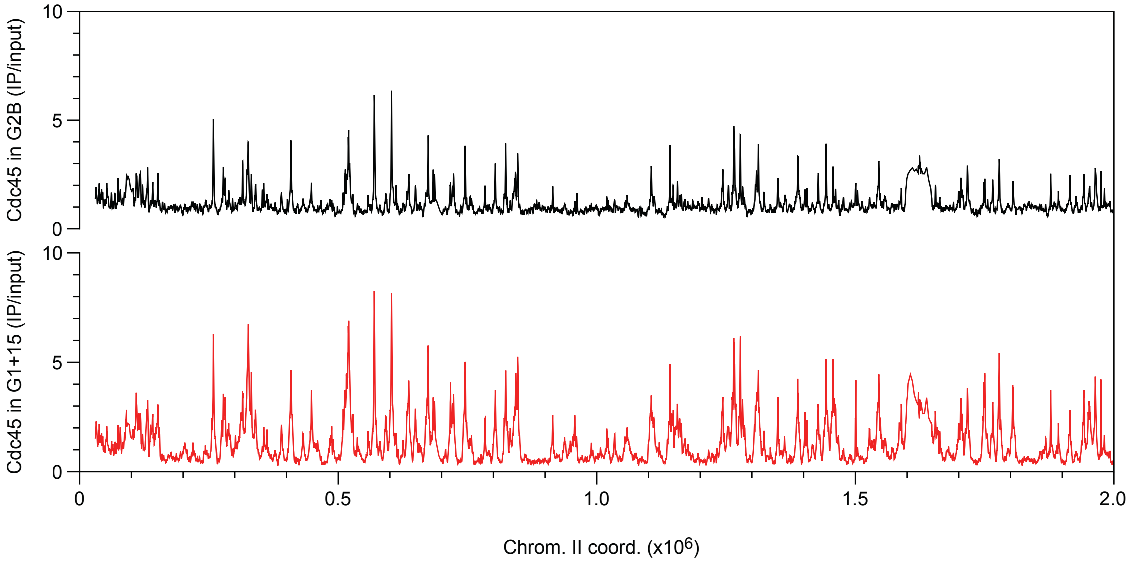

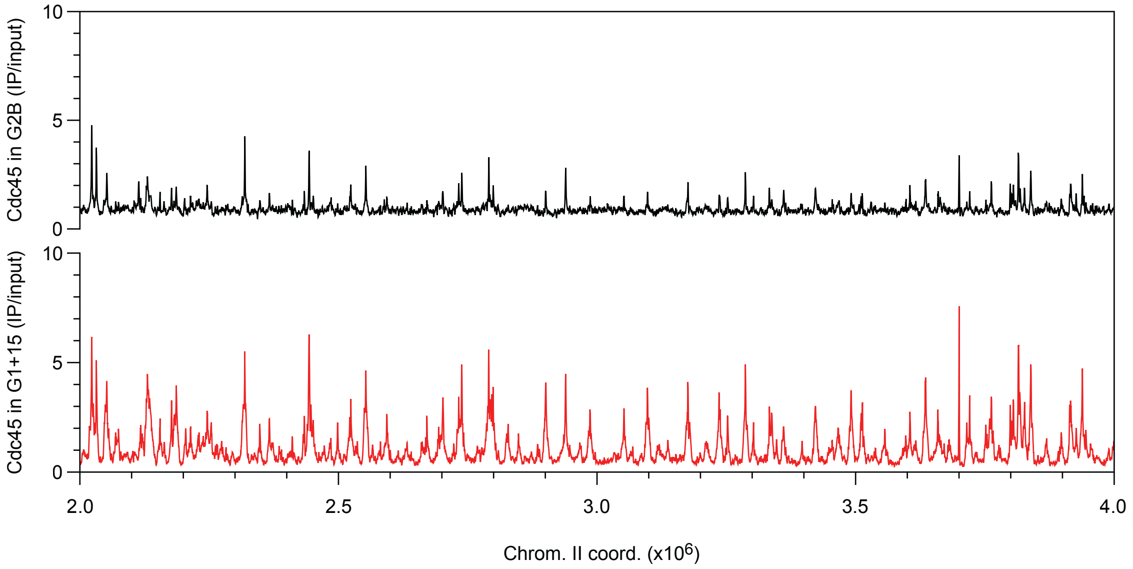

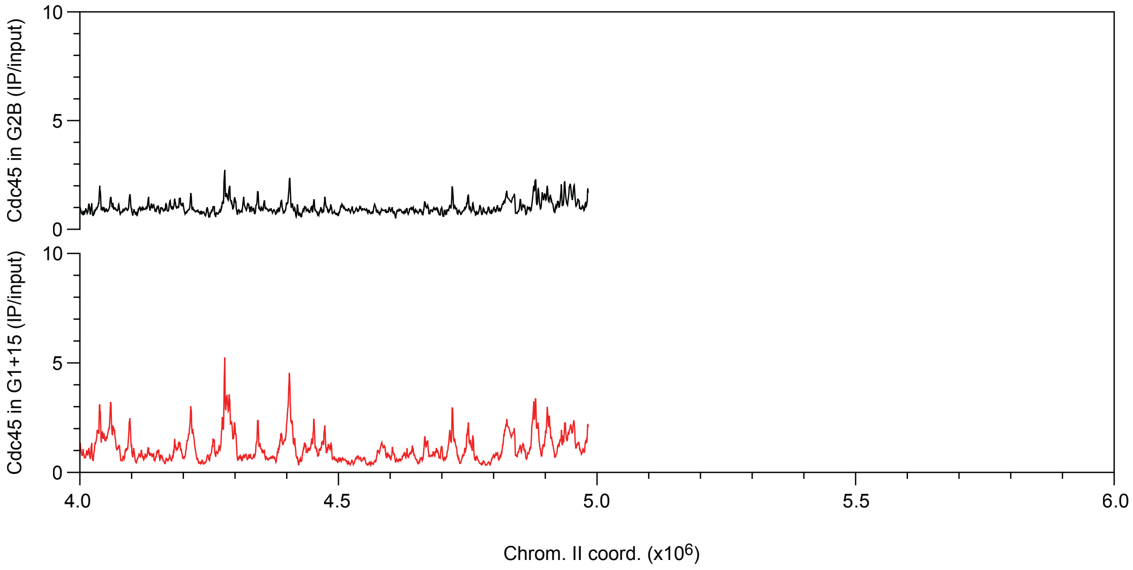

Figure S3

E_III

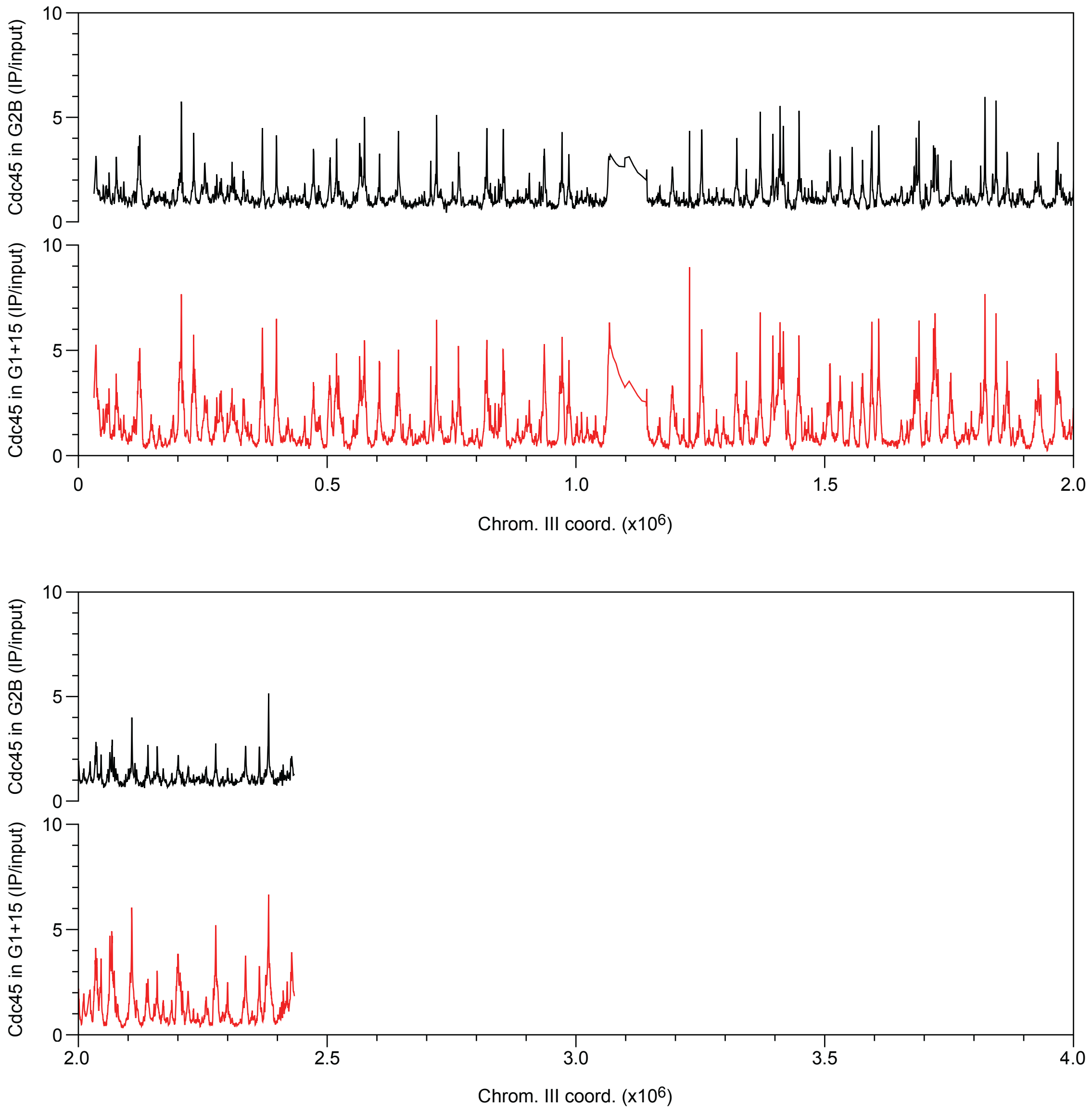

F

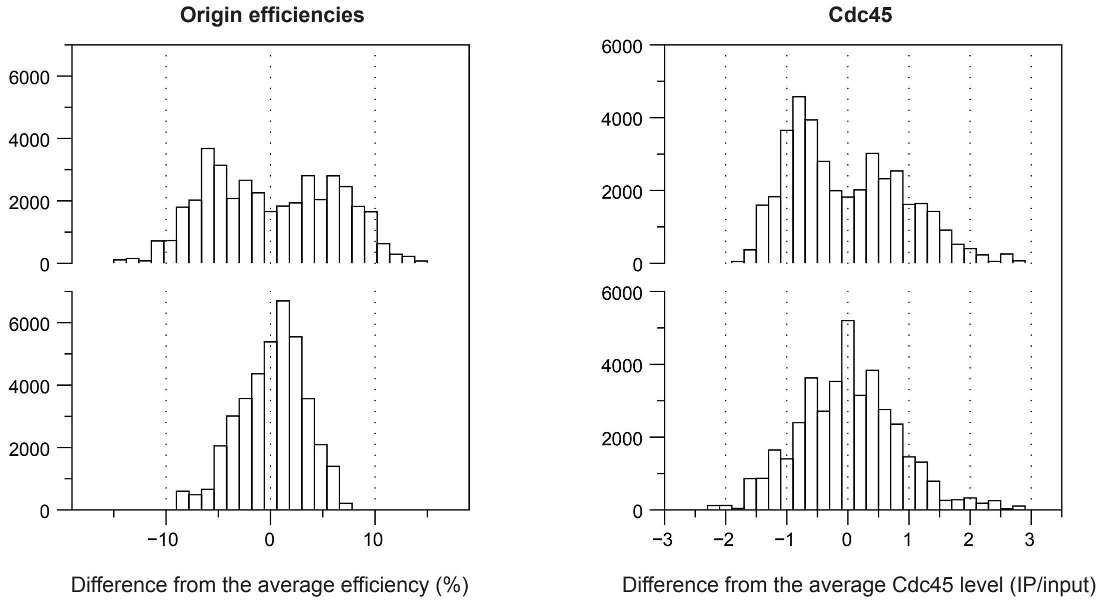

Fig S3. Genome-wide alterations in Cdc45 binding following a short G1 extension. A) Profiles of origin efficiencies (black, top) vs. Cdc45 binding (red, bottom) for G2B. x-axis: chromosome coordinates; top y-axis: origin efficiency; bottom y-axis: Cdc45 level (IP/input). Origin efficiency data are as in Fig 2C and Fig S2A. **B)** Detailed views of origin efficiencies (black, right y-axis) and Cdc45 binding (red, left y-axis) in representative regions of the genome for G2B. x-axis: chromosome coordinates. **C)** Profiles of origin efficiencies (black, top) vs. Cdc45 binding (red, bottom) for G1+15. x-axis: chromosome coordinates; top y-axis: origin efficiency; bottom y-axis: Cdc45 level (IP/input). Origin efficiency data are as in Fig 2C and Fig S2A. **D)** Detailed views of origin efficiencies (black, right y-axis) and Cdc45 binding (red, left y-axis) in representative regions of the genome for G1+15. x-axis: chromosome coordinates. **EI-III)** Detailed views of Cdc45 binding in G2B (black, top panels, as in *A*) and G1+15 (red, bottom panels, as in *C*). x-axis: chromosome coordinates; y-axis: Cdc45 level (IP/input). **F)** Histograms displaying the deviations of Cdc45 binding and origin efficiency from their genome-wide averages in G2B and G1+15. For each parameter, the average of the regional profile was calculated, and the deviation of each point in the regional profile from the corresponding mean was determined and plotted. The bimodal distributions in G2B represent efficient and inefficient regions; this is not observed for G1+15, where efficiencies between regions are more similar. This analysis shows a clear equalization of both Cdc45 and origin efficiencies in G1+15 compared to G2B.
