# Supplementary material for: CDK activity provides temporal and quantitative cues for organizing genome duplication
Source: PLoS Genet. 2018 Feb 21;14(2):e1007214. doi: 10.1371/journal.pgen.1007214 (PMC5821308; doi:10.1371/journal.pgen.1007214)
Supplement: S4 Fig — AI-III) Detailed view of the origin usage profiles of G2B (black), G1+15 (blue), and G1+165 (green) as in Fig 4C. x-axis: chromosome coordinates, y-axis: origin efficiencies. B) Pairwise comparisons of origin efficiencies in the different G1 extensions. Left panel: x-axis: efficiencies in G2B, y-axis: efficiencies in G1+165; right panel: x-axis: efficiencies in G+15, y-axis: efficiencies in G1+165. Each dot represents an origin. The dashed black lines represent efficiencies if they were identical in the two compared backgrounds. C) Origin usage characteristics in the G2B and G1 extension conditions. D) Time courses of chromatin immunoprecipitation of Cdc45 in G1+165. Note that these experiments were performed as in Fig 4A but without the addition of HU to allow progression through S phase. Efficiencies of the origins analyzed in the G1+165 condition are as follows: ori2084 (ori2004): 55%, oriJW1072: 33%, oriJW1088: 50%. x-axis: time after release from G1 arrest; y-axis: Cdc45 binding (% IP). n = 2, a representative experiment is displayed. (PDF) [file pgen.1007214.s004.pdf]

Figure S4

A_I

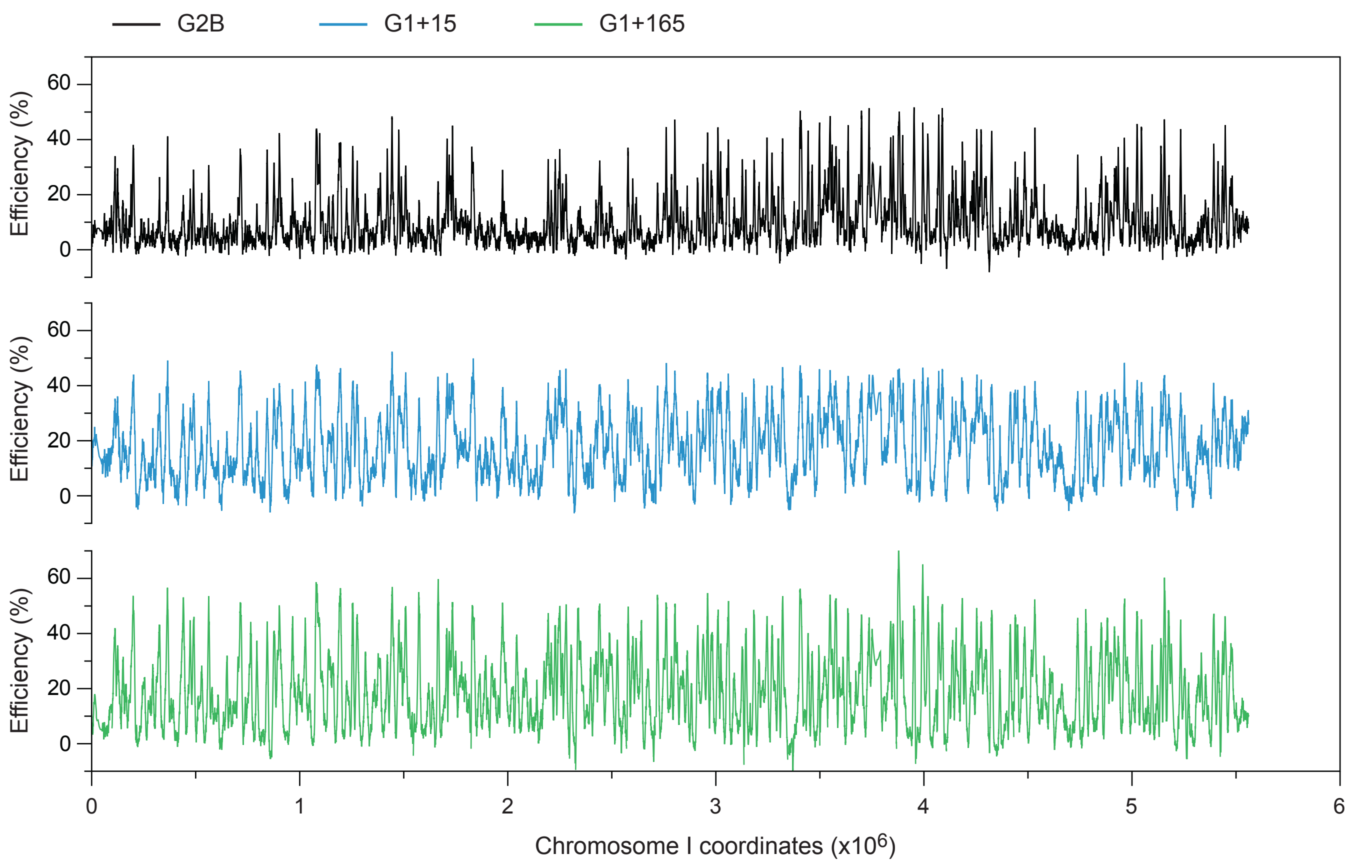

A_II

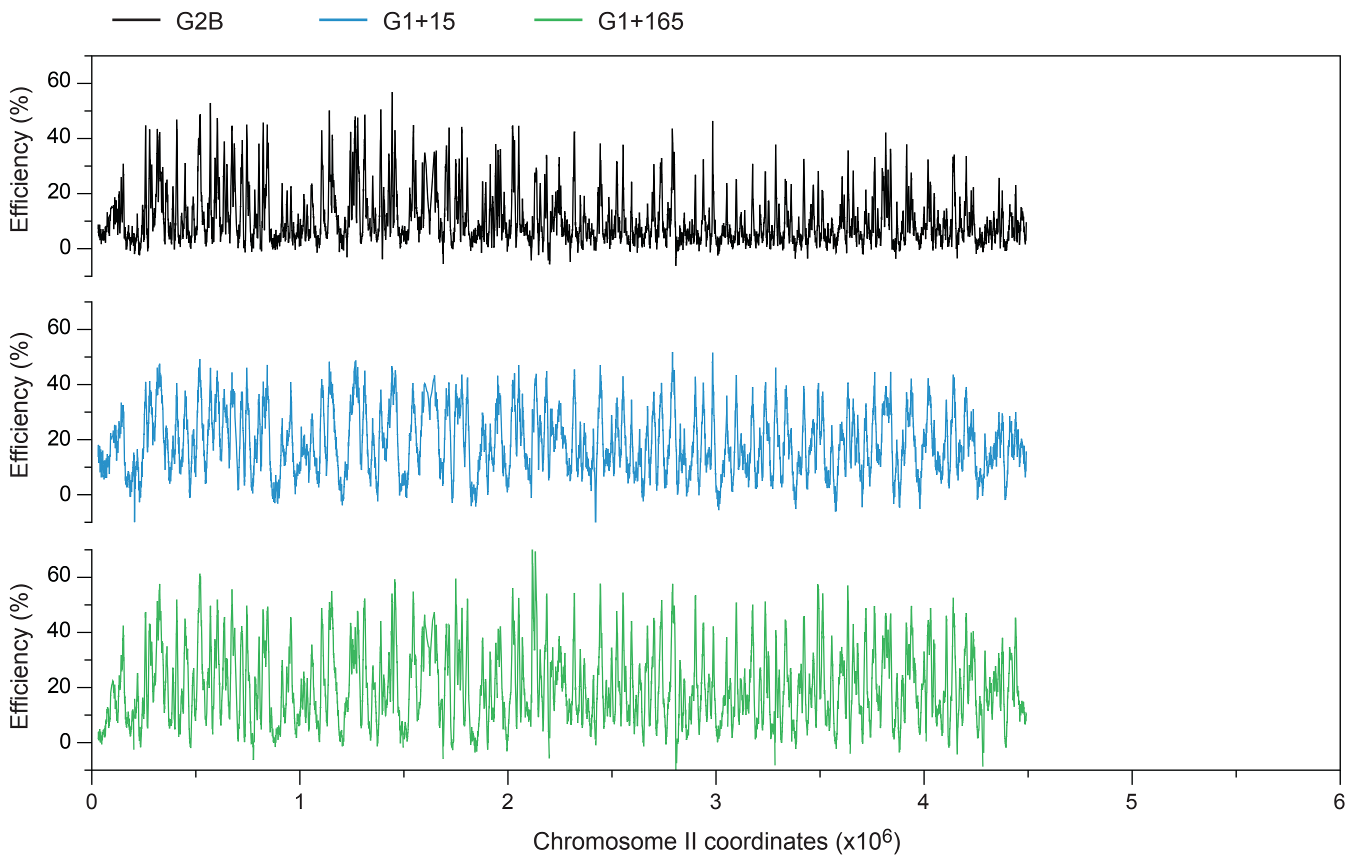

Figure S4

A_III

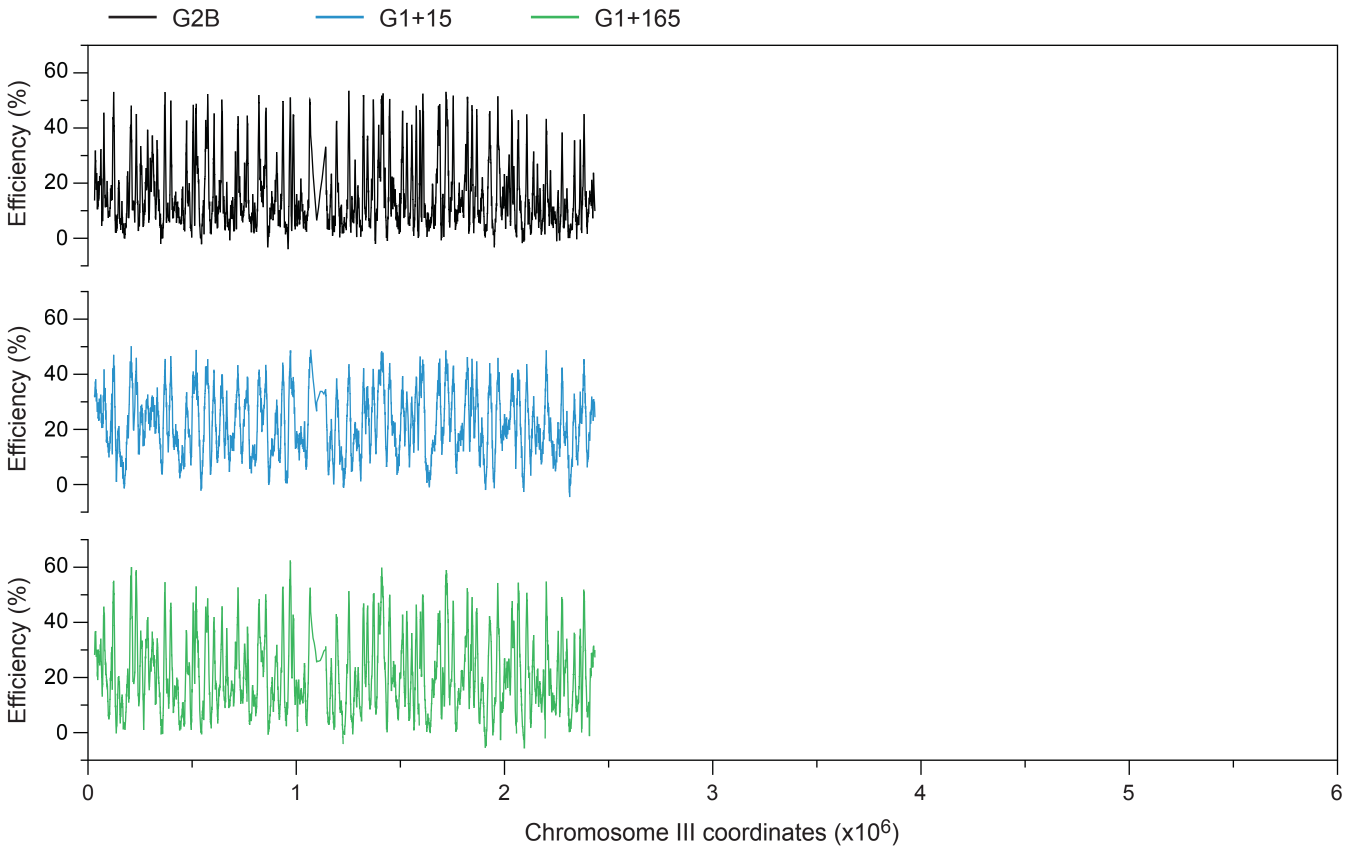

B

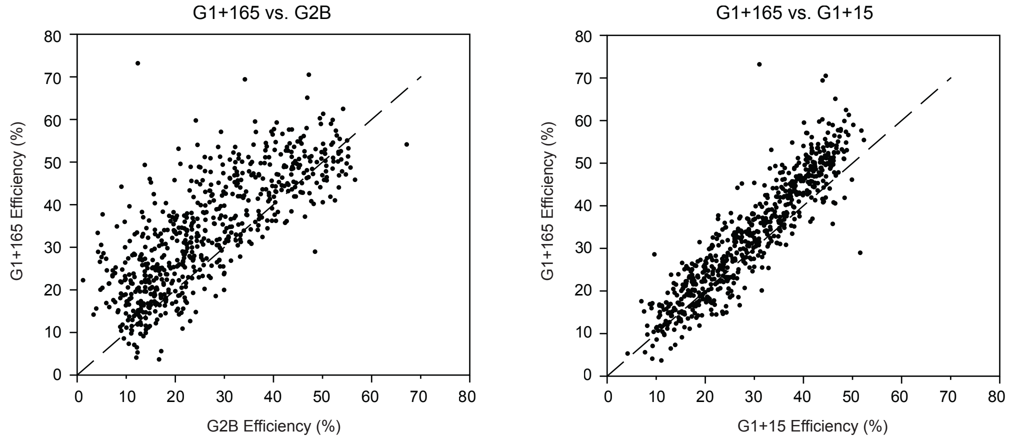

C

	G2B	G1+5	G1+15	G1+165
Total number of origins	670	670	670	670
Average origin efficiency (%)	26.4	31.9	29.4	33.4
Average efficiency difference from G2B (%)	--	5.6	3.1	7

D

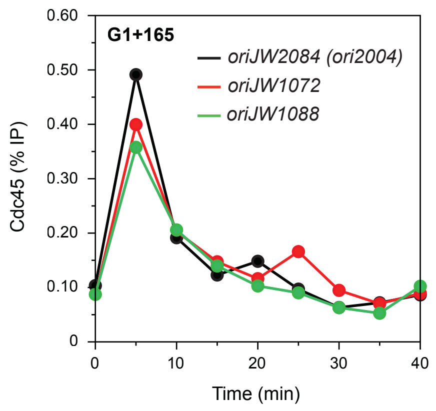

Fig S4. Comparison of replication origin usage in short and long G1 extensions. AI-III) Detailed view of the origin usage profiles of G2B (black), G1+15 (blue), and G1+165 (green) as in Fig 4C. x-axis: chromosome coordinates, y-axis: origin efficiencies. **B)** Pairwise comparisons of origin efficiencies in the different G1 extensions. Left panel: x-axis: efficiencies in G2B, y-axis: efficiencies in G1+165; right panel: x-axis: efficiencies in G+15, y-axis: efficiencies in G1+165. Each dot represents an origin. The dashed black lines represent efficiencies if they were identical in the two compared backgrounds. **C)** Origin usage characteristics in the G2B and G1 extension conditions. **D)** Time courses of chromatin immunoprecipitation of Cdc45 in G1+165. Note that these experiments were performed as in Fig 4A but without the addition of HU to allow progression through S phase. Efficiencies of the origins analyzed in the G1+165 condition are as follows: *ori2084* (*ori2004*): 55%, *oriJW1072*: 33%, *oriJW1088*: 50%. x-axis: time after release from G1 arrest; y-axis: Cdc45 binding (% IP). *n*=2, a representative experiment is displayed.
