# Supplementary material for: CDK activity provides temporal and quantitative cues for organizing genome duplication
Source: PLoS Genet. 2018 Feb 21;14(2):e1007214. doi: 10.1371/journal.pgen.1007214 (PMC5821308; doi:10.1371/journal.pgen.1007214)
Supplement: S5 Fig — AI-III) Detailed view of the origin usage profiles of S1 (black), S2.5 (red), S4 (blue), and S6 (purple) as in Fig 5B. x-axis: chromosome coordinates, y-axis: origin efficiencies. B) Pairwise comparisons of origin efficiencies in cells entering S phase with different levels of CDK activity. The dashed line represents efficiencies if they were identical in two compared backgrounds. Black dots: S2.5 vs. S1; red dots: S4 vs. S1; blue dots: S6 vs. S1. x- and y-axes: origin efficiencies in the indicated conditions. Each dot represents an origin. C) Origin usage characteristics for S1, S2.5, S4, and S6. D) DNA combing analysis of interorigin distances (IOD) in the S1 and S6 conditions. For S1, 281 IODs were analyzed in 30 independent fibers totaling 8351 kb. For S6, 242 IODs were analyzed in 32 independent fibers totaling 10539 kb. Box and whiskers plot, with outliers represented by circles. For ease of visualization, IODs > 100 kb were not displayed in the graph. This represents only 3 points in S1 (out of 281) and 7 points in S6 (out of 242). S1 has a median IOD of 15.2 kb, and S6 has a median IOD of 17.8 kb. This is consistent with a decrease in origin usage in S6 (average efficiency = 23.4%) compared to S1 (average efficiency = 33.4%). Independent-samples T-tests (two-sided) showed that these IOD values were significantly different (**: p-value = 0.0029). These results support the overall decrease in origin usage observed in our population, genome-wide analyses when cells enter S phase with lower levels of CDK activity. E) Pairwise comparisons of origin efficiencies in S2.5 vs. G1+15. The dashed black line represents efficiencies if they were identical in the two compared backgrounds. x- and y-axes: efficiencies in the indicated conditions. Each dot represents an origin. F) Regional analysis of origin efficiencies in individual replicate experiments in the S1, S2.5, S4, and S6 conditions. Average origin efficiencies were determined in continuous windows of 1000 probes [file pgen.1007214.s005.pdf]

Figure S5

A_I

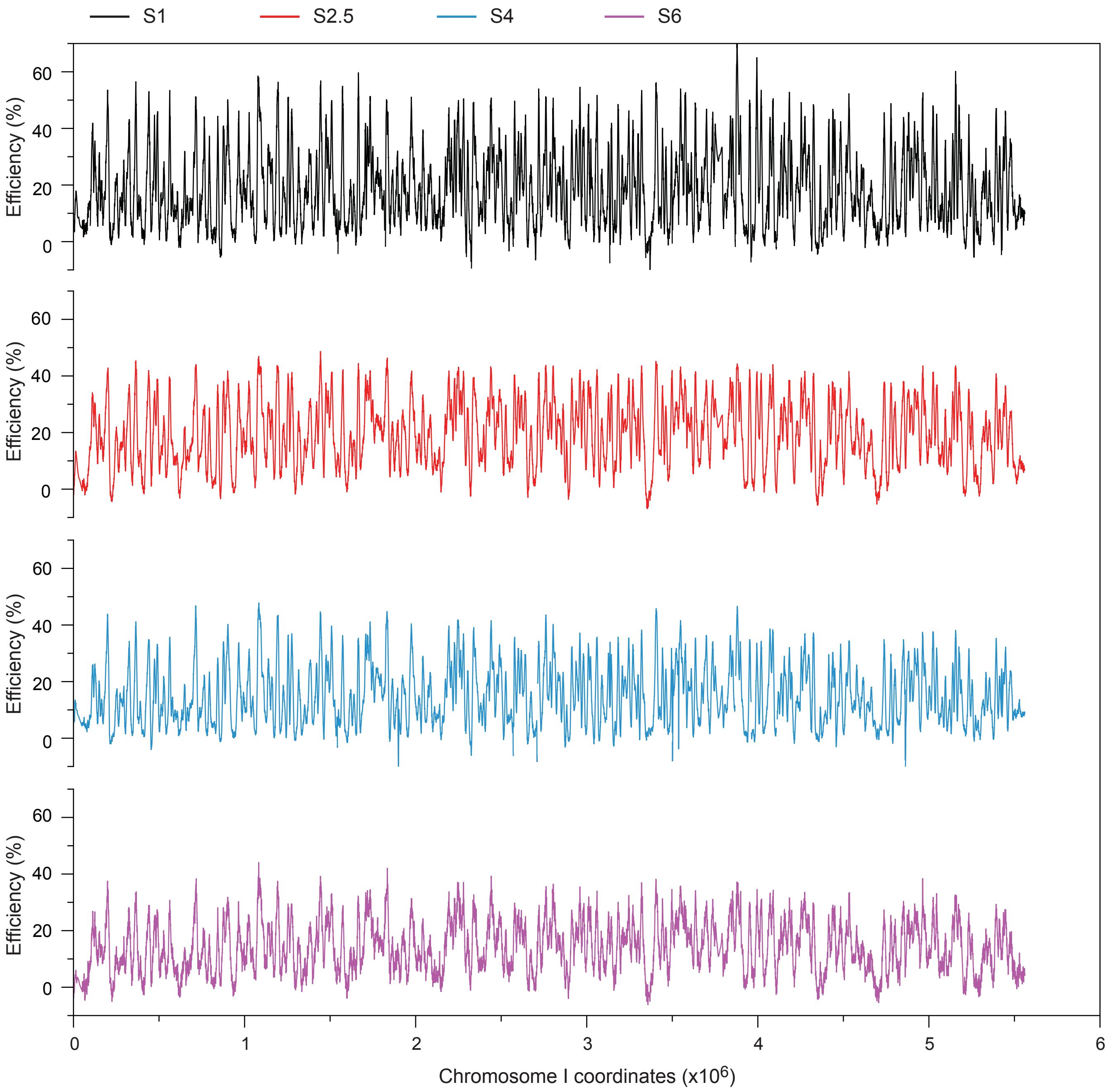
S1
S2.5
S4
S6
Efficiency (%)
60
40
20
0
Efficiency (%)
60
40
20
0
Efficiency (%)
60
40
20
0
Efficiency (%)
60
40
20
0
0
1
2
3
4
5
6
Chromosome I coordinates (x10^6)

Figure S5

A_II

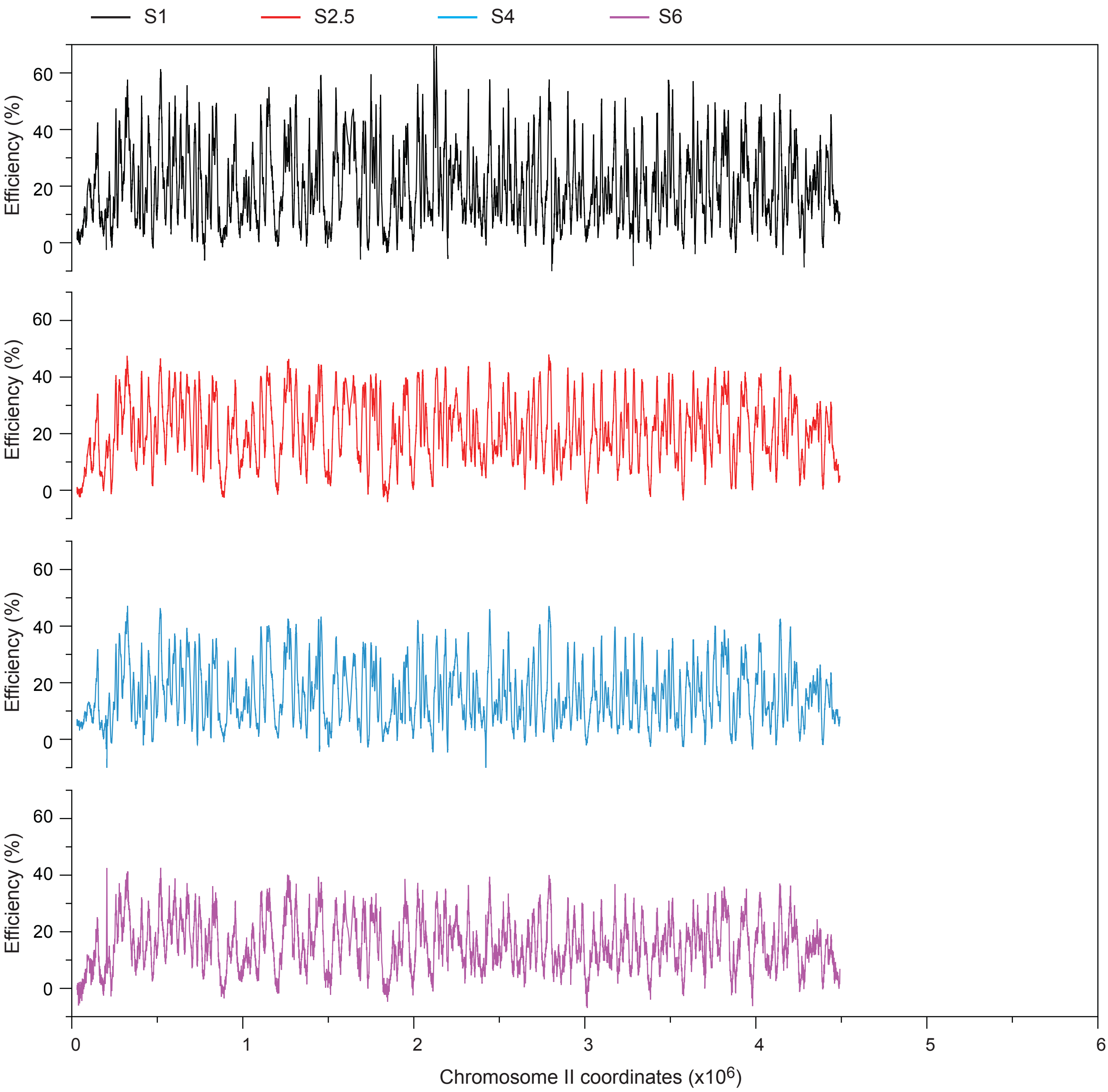

Figure S5

A_III

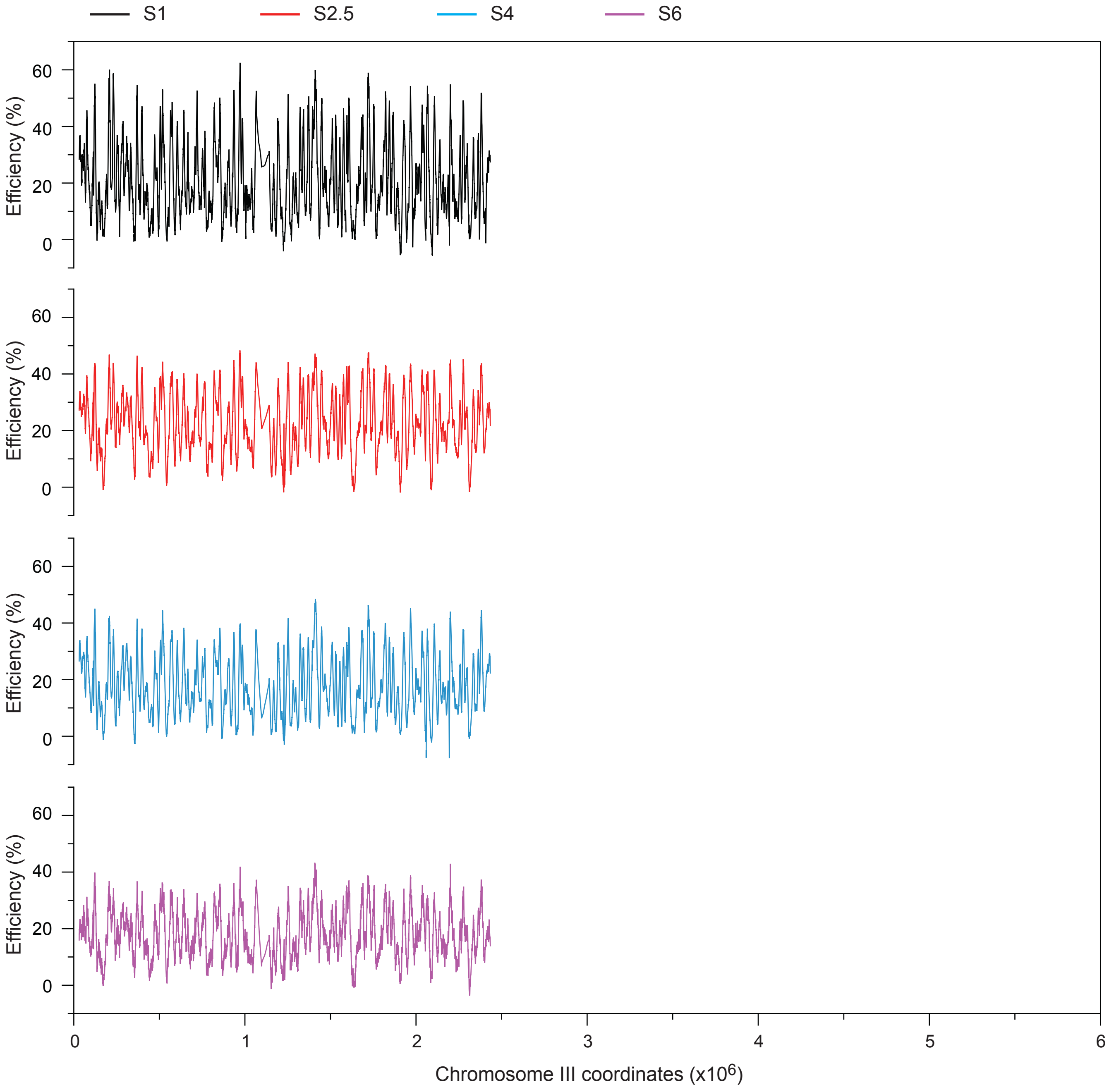

Figure S5

B

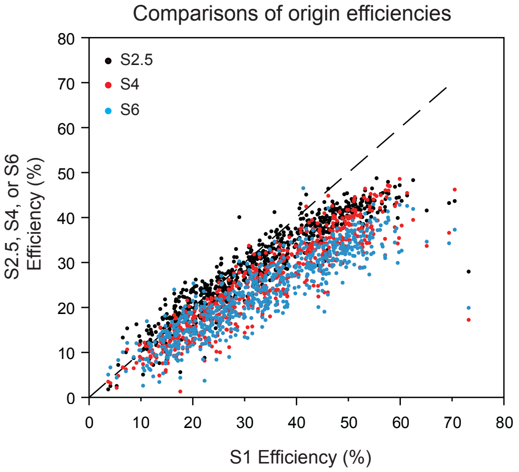

C

	S1	S2.5	S4	S6
Total number of origins	670	670	670	670
Average origin efficiency (%)	33.4	29.8	25.2	23.4
Average efficiency difference from S1 (%)	--	-3.6	-8.2	-10

D

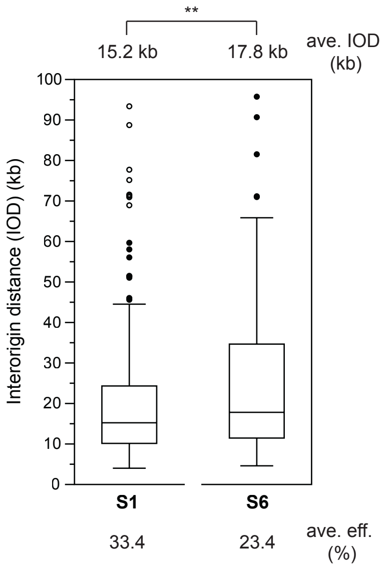

E

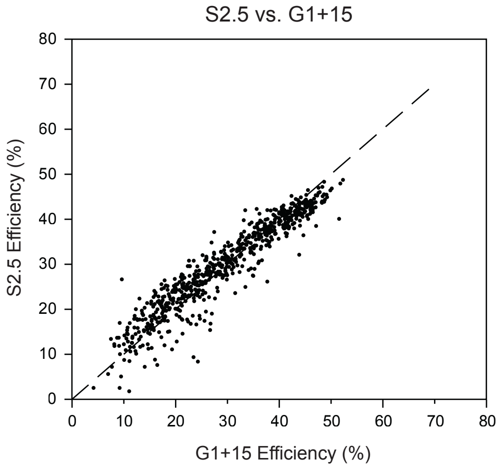

F

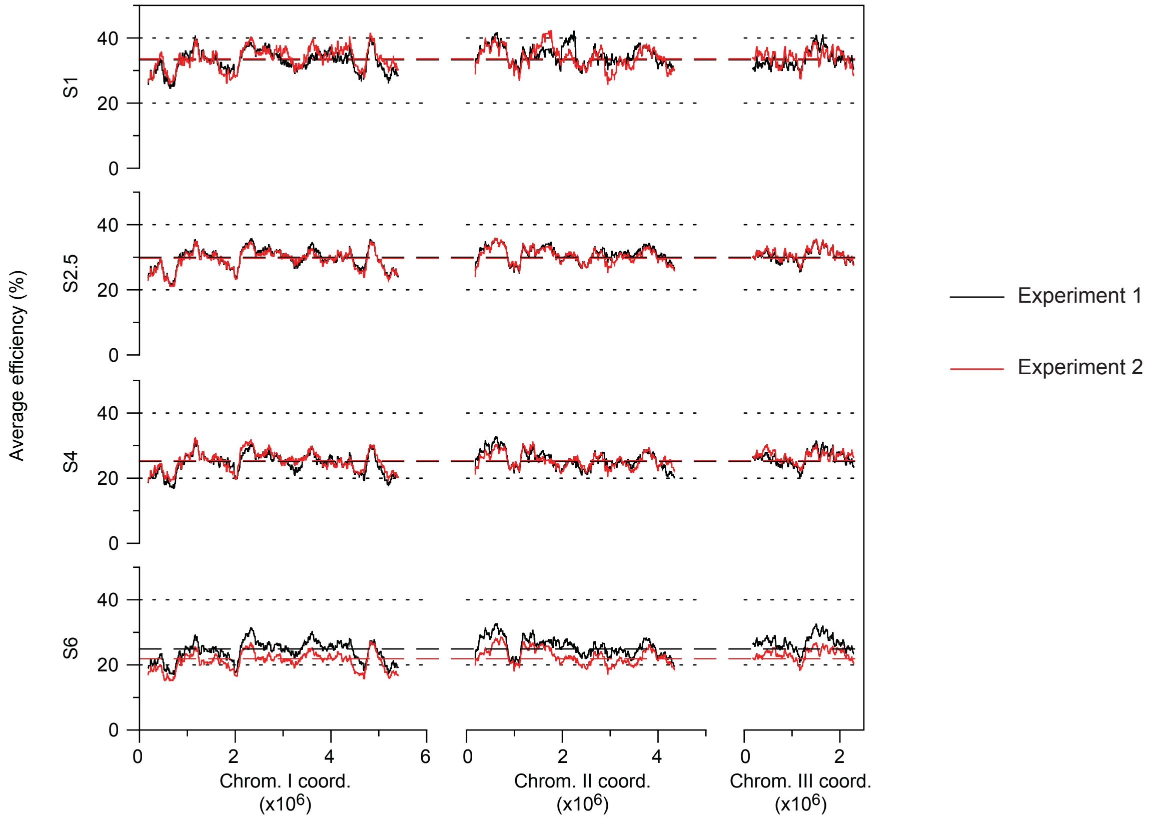

Fig S5. Replication origin usage in cells that enter S phase with different levels of CDK activity. AI-III) Detailed view of the origin usage profiles of S1 (black), S2.5 (red), S4 (blue), and S6 (purple) as in Fig 5B. x-axis: chromosome coordinates, y-axis: origin efficiencies. **B)** Pairwise comparisons of origin efficiencies in cells entering S phase with different levels of CDK activity. The dashed line represents efficiencies if they were identical in two compared backgrounds. Black dots: S2.5 vs. S1; red dots: S4 vs. S1; blue dots: S6 vs. S1. x- and y-axes: origin efficiencies in the indicated conditions. Each dot represents an origin. **C)** Origin usage characteristics for S1, S2.5, S4, and S6. **D)** DNA combing analysis of interorigin distances (IOD) in the S1 and S6 conditions. For S1, 281 IODs were analyzed in 30 independent fibers totaling 8351 kb. For S6, 242 IODs were analyzed in 32 independent fibers totaling 10539 kb. Box and whiskers plot, with outliers represented by circles. For ease of visualization, IODs > 100 kb were not displayed in the graph. This represents only 3 points in S1 (out of 281) and 7 points in S6 (out of 242). S1 has a median IOD of 15.2 kb, and S6 has a median IOD of 17.8 kb. This is consistent with a decrease in origin usage in S6 (average efficiency = 23.4%) compared to S1 (average efficiency = 33.4%). Independent-samples T-tests (two-sided) showed that these IOD values were significantly different (**: p-value = 0.0029). These results support the overall decrease in origin usage observed in our population, genome-wide analyses when cells enter S phase with lower levels of CDK activity. **E)** Pairwise comparisons of origin efficiencies in S2.5 vs. G1+15. The dashed black line represents efficiencies if they were identical in the two compared backgrounds. x- and y-axes: efficiencies in the indicated conditions. Each dot represents an origin. **F)** Regional analysis of origin efficiencies in individual replicate experiments in the S1, S2.5, S4, and S6 conditions. Average origin efficiencies were determined in continuous windows of 1000 probes (~250 kb). Black: experiment 1, red: experiment 2. Dashed line indicates the mean efficiency of all origins in each experiment; the corresponding experiment is indicated by the color. Dotted lines mark 20% and 40% efficiency. The average efficiency values are as follows: S1: 33.3% and 33.6%; S2.5: 30.0% and 29.7%; S4: 25.1% and 25.4%; S6: 24.9% and 21.9%. x-axis: chromosome coordinates, y-axis: average origin

efficiencies. Note that the repeats of S6 are detectably different (see also Table S2). Indeed, the low level of CDK activity in cells just after release from G1 in this condition may be just above the threshold for S phase entry, which may render them more sensitive to small variations in experimental conditions.
