# Supplementary material for: CDK activity provides temporal and quantitative cues for organizing genome duplication
Source: PLoS Genet. 2018 Feb 21;14(2):e1007214. doi: 10.1371/journal.pgen.1007214 (PMC5821308; doi:10.1371/journal.pgen.1007214)
Supplement: S6 Fig — A-J) Graphs comparing the two repeats of origin mapping experiments performed in all conditions in this study. The Spearmans’ rank correlation coefficient (ρ) for each comparison is displayed. ***: p < 0.001. The dashed black lines represent efficiencies if they were identical in the two repeats. x- and y-axes: origin efficiencies in the indicated conditions. These results show the high level of reproducibility of our datasets. (PDF) [file pgen.1007214.s006.pdf]

Figure S6

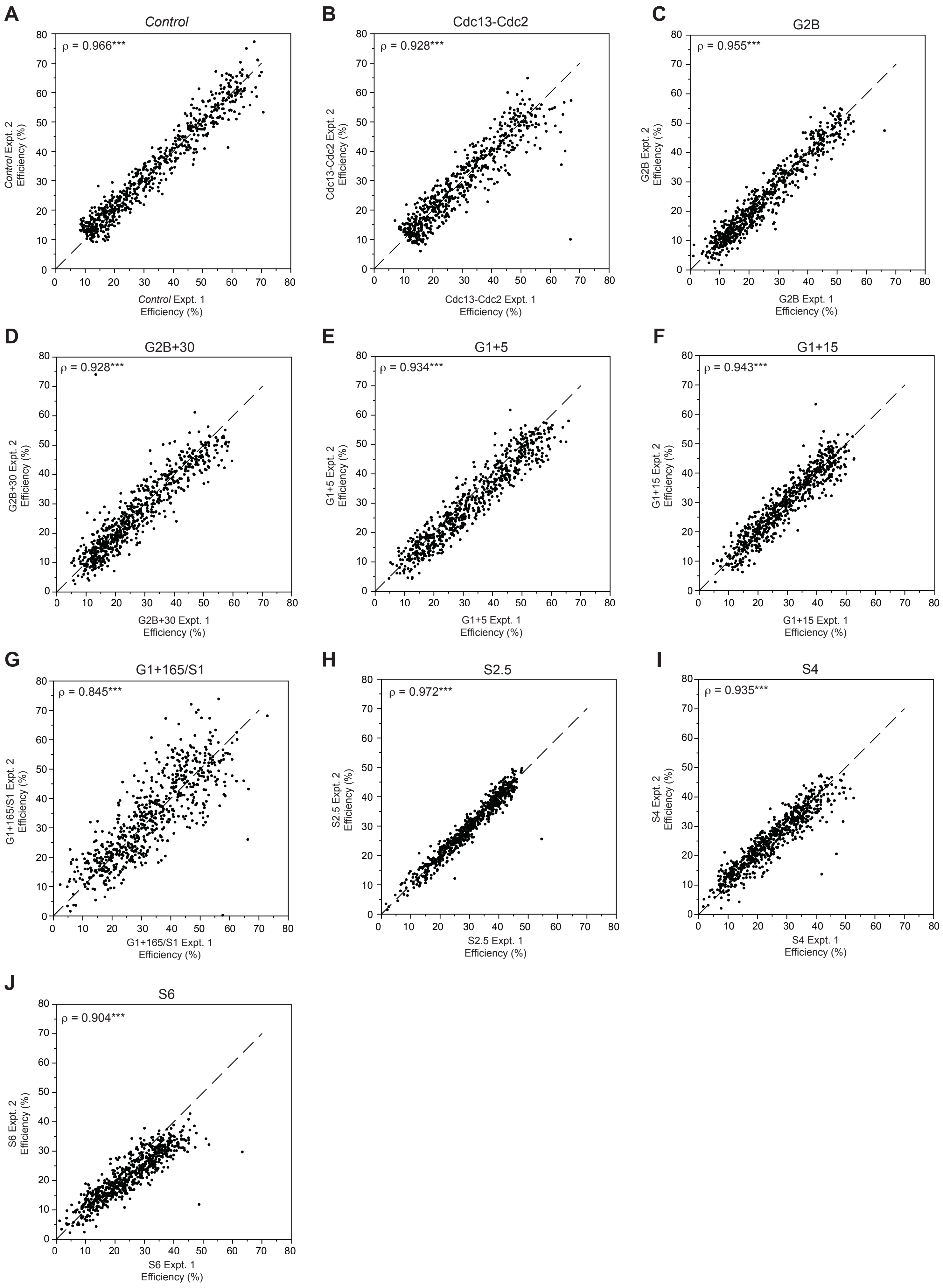

A
Control
ρ = 0.966***
Control Expt. 2 Efficiency (%)
Control Expt. 1 Efficiency (%)
B
Cdc13-Cdc2
ρ = 0.928***
Cdc13-Cdc2 Expt. 2 Efficiency (%)
Cdc13-Cdc2 Expt. 1 Efficiency (%)
C
G2B
ρ = 0.955***
G2B Expt. 2 Efficiency (%)
G2B Expt. 1 Efficiency (%)
D
G2B+30
ρ = 0.928***
G2B+30 Expt. 2 Efficiency (%)
G2B+30 Expt. 1 Efficiency (%)
E
G1+5
ρ = 0.934***
G1+5 Expt. 2 Efficiency (%)
G1+5 Expt. 1 Efficiency (%)
F
G1+15
ρ = 0.943***
G1+15 Expt. 2 Efficiency (%)
G1+15 Expt. 1 Efficiency (%)
G
G1+165/S1
ρ = 0.845***
G1+165/S1 Expt. 2 Efficiency (%)
G1+165/S1 Expt. 1 Efficiency (%)
H
S2.5
ρ = 0.972***
S2.5 Expt. 2 Efficiency (%)
S2.5 Expt. 1 Efficiency (%)
I
S4
ρ = 0.935***
S4 Expt. 2 Efficiency (%)
S4 Expt. 1 Efficiency (%)
J
S6
ρ = 0.904***
S6 Expt. 2 Efficiency (%)
S6 Expt. 1 Efficiency (%)
0
10
20
30
40
50
60
70
80

Fig S6. Comparisons of origin efficiencies from individual experiments for each condition. A-J) Graphs comparing the two repeats of origin mapping experiments performed in all conditions in this study. The Spearmans' rank correlation coefficient (ρ) for each comparison is displayed. ***: $p < 0.001$. The dashed black lines represent efficiencies if they were identical in the two repeats. x- and y-axes: origin efficiencies in the indicated conditions. These results show the high level of reproducibility of our datasets.
