# Supplementary material for: CDK activity provides temporal and quantitative cues for organizing genome duplication
Source: PLoS Genet. 2018 Feb 21;14(2):e1007214. doi: 10.1371/journal.pgen.1007214 (PMC5821308; doi:10.1371/journal.pgen.1007214)
Supplement: S2 Table — Independent-samples T-tests (two-sided) were applied. Statistically significant differences are shown in red. (DOCX) [file pgen.1007214.s008.docx]

**Table S2**: Statistical analyses of the differences in origin usage between two conditions. An independent-samples T-test (two-sided) was applied. Statistically significant differences are shown in red.

| **Comparisons between conditions** |  |  |  |
| --- | --- | --- | --- |
|  | **p-value** | **t-value** | **Degrees of freedom** |
| *Control* vs Cdc13-Cdc2 | 4.70e-07 | 5.0649 | 1251.511 |
| G2B vs. G1+5 | < 2.2e-16 | -9.5207 | 1333.406 |
| G2B vs. G1+15 | 1.74e-11 | -6.7869 | 1301.192 |
| G2B vs. G1+165 | < 2.2e-16 | -11.7355 | 1336.769 |
| G1+15 vs. G1+165 | 2.79e-09 | -5.9857 | 1287.756 |
| S1 vs. S2.5 | 2.68e-08 | 5.5972 | 1233.256 |
| S1 vs. S4 | < 2.2e-16 | 12.6571 | 1240.339 |
| S1 vs. S6 | < 2.2e-16 | 16.1642 | 1151.679 |
| S2.5 vs. S4 | < 2.2e-16 | 8.4004 | 1337.814 |
| S2.5 vs. S6 | < 2.2e-16 | 12.4965 | 1317.253 |
| G2B vs. G2B+30 | 0.0014^1^ | -3.2014 | 1337.81 |
|  |  |  |  |
| **Comparisons between repeats** | **p-value** | **t-value** | **Degrees of freedom** |
| *Control* | 0.5996 | -0.5251 | 1315.548 |
| Cdc13-Cdc2 | 0.4706 | 0.7218 | 1241.638 |
| G2B | 0.1872 | 1.3195 | 1337.596 |
| G2B+30 | 0.3044 | 1.0275 | 1337.571 |
| G1+5 | 0.003339 | 2.94 | 1332.340 |
| G1+15 | 0.8633 | 0.1723 | 1337.205 |
| G1+165/S1 | 0.6701 | -0.4261 | 1337.941 |
| S2.5 | 0.6291 | -0.4832 | 1337.855 |
| S4 | 0.5792 | -0.5547 | 1327.637 |
| S6 | 1.32e-09^2^ | 6.1103 | 1269.267 |
|  |  |  |  |

1: Comparison of the G2B vs. G2B+30 origin efficiencies shows that these programs are not significantly different (see also Fig. S2C). This demonstrates that maintaining cells in S phase in the presence of HU for an additional 30 min does not lead to an alteration in origin efficiencies.

2: Note that the two repeats of S6 show detectable differences. This may be due in part to the low level of CDK activity present as cells begin S phase in these conditions, which is likely to be just above the threshold for S phase entry. Cells may then be more sensitive to small variations in inhibitor concentration or experimental conditions.
